# Asexual expansion of *Toxoplasma gondii* merozoites is distinct from tachyzoites and entails expression of non-overlapping gene families to attach, invade, and replicate within feline enterocytes

**DOI:** 10.1186/s12864-015-1225-x

**Published:** 2015-02-13

**Authors:** Adrian B Hehl, Walter U Basso, Christoph Lippuner, Chandra Ramakrishnan, Michal Okoniewski, Robert A Walker, Michael E Grigg, Nicholas C Smith, Peter Deplazes

**Affiliations:** Institute of Parasitology-University of Zurich, Winterthurerstrasse 266a, Zürich, 8057 Switzerland; Current address: Department of Anaesthesiology and Pain Medicine, Inselspital, University of Bern, Freiburgstrasse, Bern, 3010 Switzerland; Functional Genomics Center Zurich, Winterthurerstrasse 190, Zürich, 8057 Switzerland; Queensland Tropical Health Alliance Research Laboratory, Faculty of Medicine, Health and Molecular Sciences, James Cook University, Cairns Campus, McGregor Road, Smithfield, QLD 4878 Australia; Molecular Parasitology Section, Laboratory of Parasitic Diseases, NIAID, NIH, Bethesda, Maryland USA

**Keywords:** *Toxoplasma gondii*, Apicomplexa, Coccidia, Cat, Enteroepithelial development, Merozoite, Schizont, Comparative transcriptomics, Surface antigen, Stage-specific gene expression

## Abstract

**Background:**

The apicomplexan parasite *Toxoplasma gondii* is cosmopolitan in nature, largely as a result of its highly flexible life cycle. Felids are its only definitive hosts and a wide range of mammals and birds serve as intermediate hosts. The latent bradyzoite stage is orally infectious in all warm-blooded vertebrates and establishes chronic, transmissible infections. When bradyzoites are ingested by felids, they transform into merozoites in enterocytes and expand asexually as part of their coccidian life cycle. In all other intermediate hosts, however, bradyzoites differentiate exclusively to tachyzoites, and disseminate extraintestinally to many cell types. Both merozoites and tachyzoites undergo rapid asexual population expansion, yet possess different effector fates with respect to the cells and tissues they develop in and the subsequent stages they differentiate into.

**Results:**

To determine whether merozoites utilize distinct suites of genes to attach, invade, and replicate within feline enterocytes, we performed comparative transcriptional profiling on purified tachyzoites and merozoites. We used high-throughput RNA-Seq to compare the merozoite and tachyzoite transcriptomes. 8323 genes were annotated with sequence reads across the two asexually replicating stages of the parasite life cycle. Metabolism was similar between the two replicating stages. However, significant stage-specific expression differences were measured, with 312 transcripts exclusive to merozoites versus 453 exclusive to tachyzoites. Genes coding for 177 predicted secreted proteins and 64 membrane- associated proteins were annotated as merozoite-specific. The vast majority of known dense-granule (*GRA*), microneme (*MIC*), and rhoptry (*ROP*) genes were not expressed in merozoites. In contrast, a large set of surface proteins (SRS) was expressed exclusively in merozoites.

**Conclusions:**

The distinct expression profiles of merozoites and tachyzoites reveal significant additional complexity within the *T. gondii* life cycle, demonstrating that merozoites are distinct asexual dividing stages which are uniquely adapted to their niche and biological purpose.

**Electronic supplementary material:**

The online version of this article (doi:10.1186/s12864-015-1225-x) contains supplementary material, which is available to authorized users.

## Background

*Toxoplasma gondii* is an intracellular zoonotic parasite that chronically infects 30% of the world’s human population [1]. It has a complex life cycle, infecting a wide range of mammals and birds as intermediate hosts but with felids as the only definitive hosts. Intermediate hosts can become infected through ingestion of oocysts shed into the environment *via* cat feces or by ingesting tissue cysts in meat or viscera [2]. During acute infection of naïve intermediate hosts, sporozoites from oocysts or bradyzoites from tissue cysts differentiate quickly into tachyzoites, which divide rapidly and disseminate throughout the host’s body. Tachyzoites are, however, controlled efficiently by cell mediated immunity and the parasite reverts to the slowly replicating or quiescent bradyzoite form, which resides within tissue cysts that are particularly abundant in brain and heart muscle but are also present throughout skeletal muscle [3]. When cats ingest tissue cysts, bradyzoites can take a different developmental pathway. They are released from their cysts and invade enterocytes of the small intestine, transforming into schizonts [4,5]. The parasite population that develops in cat enterocytes undergoes a classical coccidian cycle involving several rounds of asexual division and amplification followed by differentiation into macro- and microgamonts, the dimorphic stages of sexual development. Microgametes fertilize macrogametes, producing diploid zygotes that subsequently develop into unsporulated oocysts that are excreted in the feces of the cat. The sexual phase continues within the oocyst as meiosis ensues, followed by mitosis to produce infectious sporozoites, encased within sporocysts inside the oocysts.

The readily culturable, rapidly dividing tachyzoite is the best studied form of *T. gondii* by far – there is abundant information about cell cycle, metabolism and host parasite interactions for this stage [6]. In contrast, the merozoite, which is the other rapidly dividing asexual form of *T. gondii* that ultimately generates hundreds of millions of gametes*,* is the least well studied developmental stage. This is largely because merozoites are not cultivatable *in vitro* and difficult to access *in vivo*. Thus, investigation of the molecular mechanisms governing initiation of parasite amplification preceding the development of sexual stages in cats has been severely hampered. The need for a more detailed understanding of the development in the definitive host is underscored by the fact that infected cats shed hundreds of millions of oocysts that can remain infectious for over a year to a wide range of highly susceptible intermediate hosts, including humans [7]. Here, we developed improved protocols for cat infection, parasite isolation, and next generation sequencing to close this knowledge gap by building a transcription profile for the merozoite stage of enteroepithelial development. Using genome-wide comparative transcriptomics, we show that merozoites express distinct gene families in a stage-specific fashion, and fail to express the majority of annotated ROP, GRA and MIC proteins which are upregulated during tachyzoite replication. Among the most highly differentially regulated parasite proteins were several large gene families, including those coding for SRS proteins found on the parasite cell surface. Other key genes expressed by tachyzoites whose products are known to facilitate motility, host seeking, attachment, invasion, and remodeling of the parasitophorous vacuole (PV) within the parasitized host cell were not expressed in merozoites. This strongly suggests that merozoites are biologically distinct and utilize a different suite of genes that are necessary for asexual expansion within feline enterocytes prior to gamont development.

## Results and discussion

### Purification of merozoites from feline enterocytes for RNA-Seq analysis

**Figure 1:**
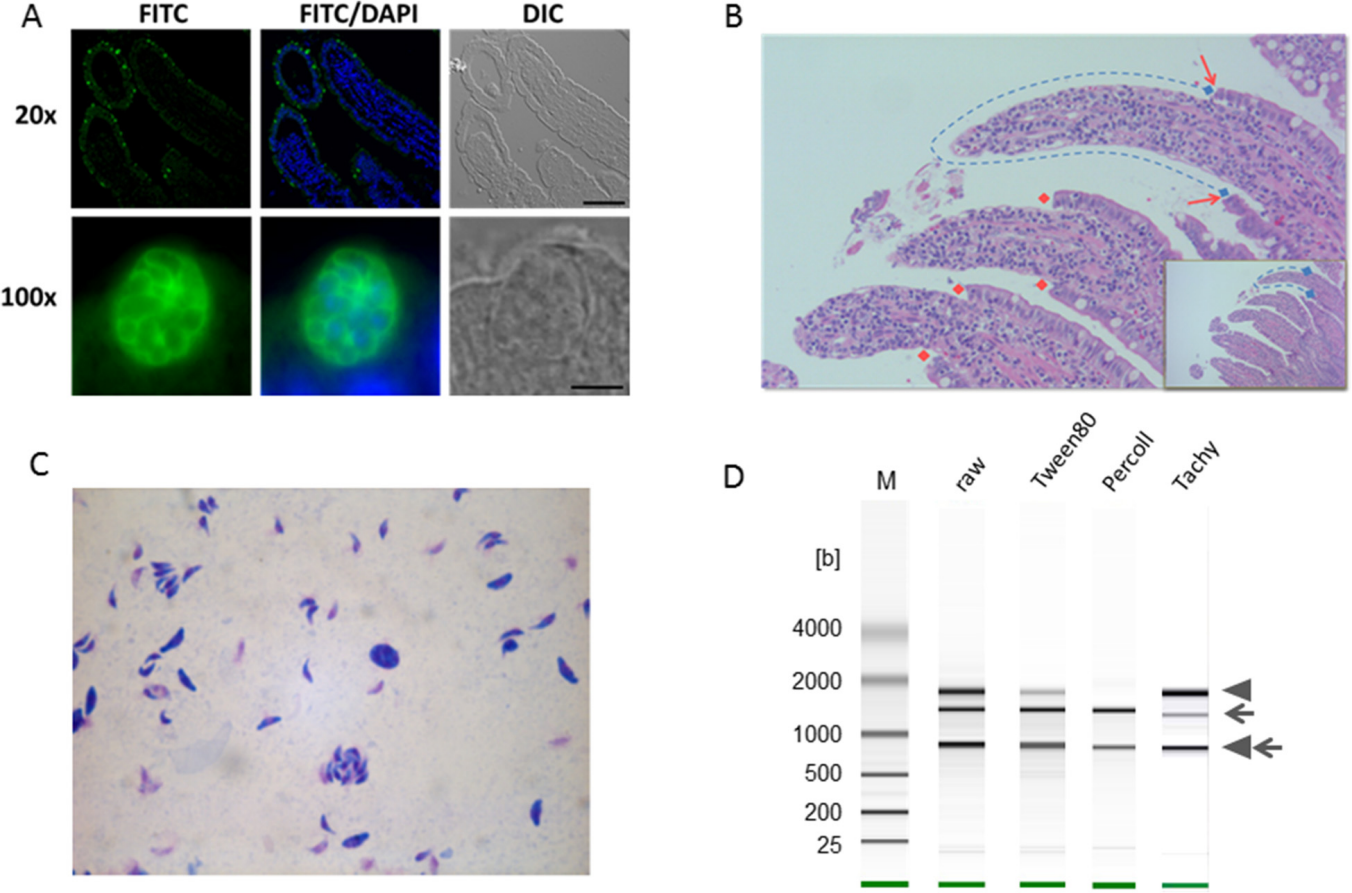
**Experimental infection, parasite isolation, and RNA preparation. A)** Immunofluorescence assay using sheep immune serum against *T. gondii* revealed numerous infected enterocytes. Nuclei are counterstained with DAPI. Shown in the top panel is a 20x magnification of a section of the small intestine (bar = 100 μM) where villi are visible. The bottom panel at 100x magnification shows a schizont containing several merozoites (scale bar = 5 μm). **B)** Enterocytes containing CZ-strain merozoite stages were stripped away selectively, leaving the villus structure and the cells of the *lamina propria* intact. Histology section (Hematoxilin & Eosin stained) showing stripped villi at day 5 post infection. **C)** Microscopic examination of parasites in the detergent washed preparation showed only merozoite stages. **D)** Quality control of total RNA extracted from parasite preparations separated on an Agilent RNA 6000 Pico Chip. The bands generated by host 28S/18S ribosomal RNA (arrowheads) and parasite 26S/18S ribosomal RNA (arrows) as well as a size marker are indicated. The samples analyzed were: raw, unprocessed material from scraped intestinal lining; Tween 80, material that was syringe-passaged and washed twice with PBS/0.05% Tween 80; Percoll, highly enriched parasite fraction after detergent treatment and Percoll gradient centrifugation; Tachy, RNA prepared from tachyzoites grown in cell culture with human foreskin fibroblasts as host cells.

*T. gondii* parasite preparations were generated from enterocytes from an infected cat at onset of patency (5d post infection). The enterocyte cell layer containing replicating parasites as shown by IFA (Figure 1A) from two regions of the rinsed and opened small intestine were selectively harvested by mechanical stripping (Figure 1B). Repeated centrifugation and re-suspension of this material with ice-cold 0.05% Tween80 in PBS yielded microscopically pure fractions of extracellular merozoites (two biological replicates). The released merozoites showed differential staining with Diff-Quick (Figure 1C); no gametocyte stages were observed in this fraction by microscopy. However, even though there is no evidence for this in the RNA-Seq data (see also in Methods) we cannot completely exclude minor contributions from sexual stages (macrogametes, microgametes) to the RNA pool selected for analysis. Nevertheless, based on the overwhelming majority of merozoites in the sample at this early time point of infection we will henceforth refer to this fraction as “merozoites”. Although further Percoll gradient purification yielded merozoites that were completely free of host material, we used the detergent-washed preparations containing minor contamination with host RNA (Figure 1D) for sequencing to avoid any changes to the parasite transcriptome due to additional manipulations.

### Merozoites possess highly significant stage-specific mRNA expression differences from tachyzoites

**Figure 2:**
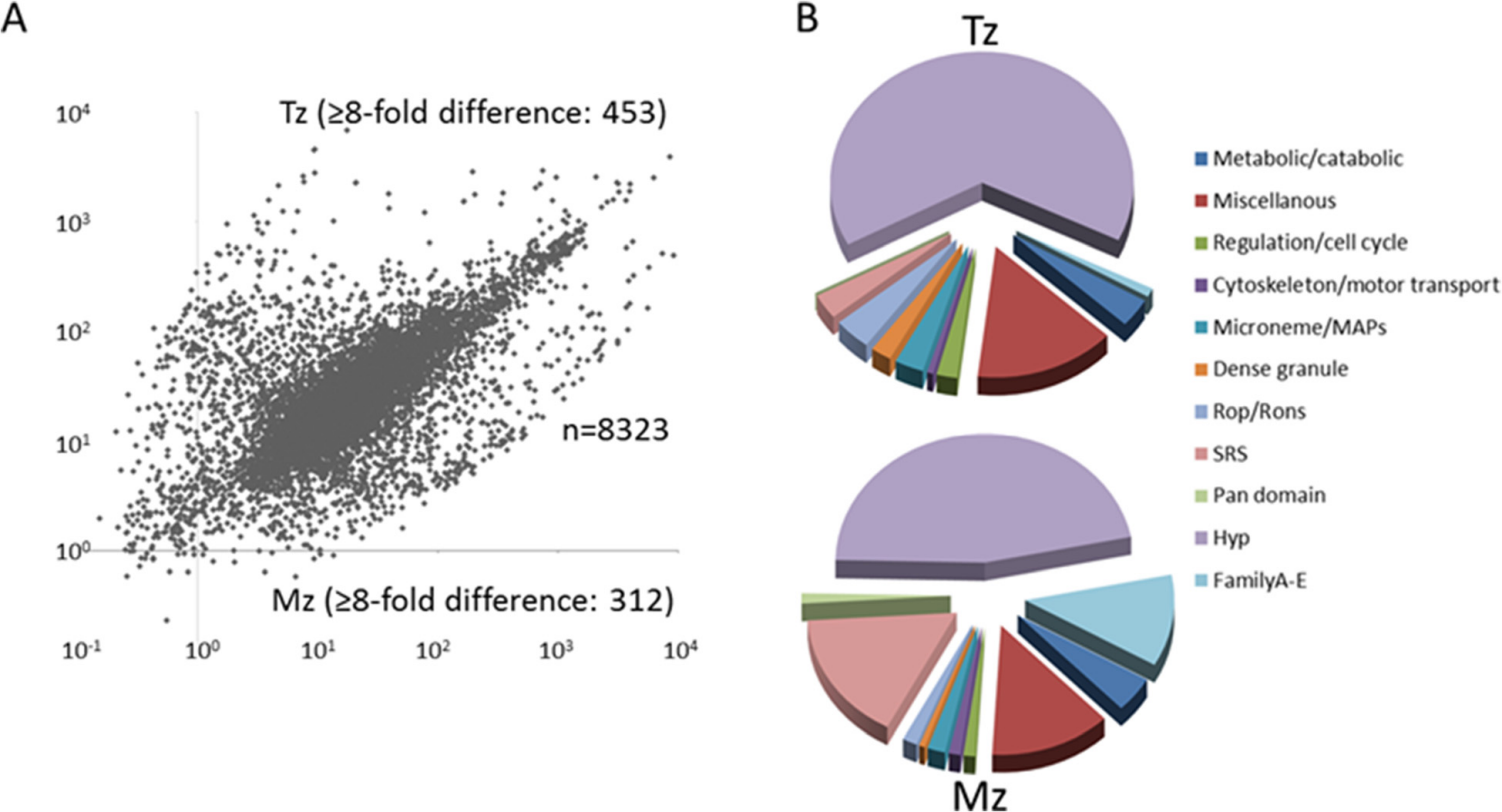
**Global comparative transcriptome analysis. A)** Scatterplot depicting expression levels as mapped read (DESeq values) of 8323 identified genes in CZ tachyzoites (Tz) and merozoites (Mz). The threshold for stage-regulated expression was set at ≥8-fold difference. **B)** Pie charts show parsing of 453 tachyzoite and 312 merozoite stage-regulated genes into functional categories.

Read mapping revealed a significant number of unique reads for 8323 genes after removal of reads mapping to tRNA and ribosomal RNA sequences, and normalization. Calculation of RPKM values (i.e. mapped reads per kilo base per million reads in dataset) to normalize for dataset size and gene length revealed 7148 genes with threshold RPKMs ≥10 in at least one of the two life-cycle stages. Comparative analysis (tachyzoite versus merozoite mapped reads per gene) was performed using DESeq [8] to detect differential gene expression. We used a high threshold of ≥ 8-fold difference in mRNA levels (measured as normalized averaged mapped sequence reads per gene from two datasets each for tachyzoites and merozoites) to identify stage-specifically regulated genes [9]. The rigorous analytical approach revealed significant stage-specific expression differences for approximately 10% of genes annotated in ToxoDB (Figure 2A). Applying the ≥ 8-fold difference threshold we identified 453 genes with tachyzoite-specific, versus 312 genes with merozoite-specific expression. This strategy therefore revealed only the most strongly stage-specifically regulated genes; maximal differences of RNA expression values of 54-fold (ORF TGME49_295662, merozoite-specific) and 404-fold (ORF TGME49_215980, tachyzoite-specific) were measured.

**Figure 3:**
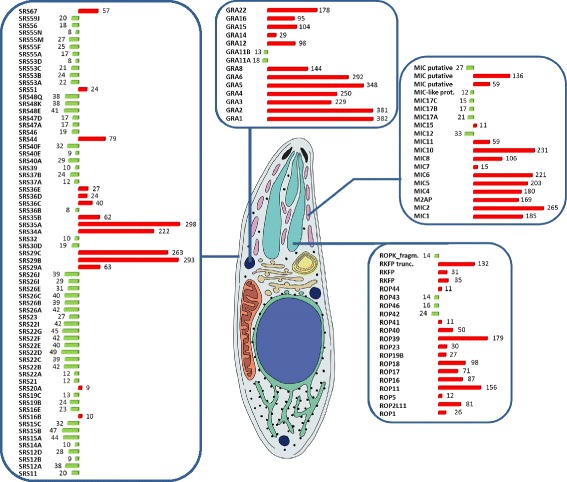
**Differential expression of** ***SRS*** **s and genes coding for secretory organelle (microneme, rhoptry, dense granule) proteins.** Bar graphs indicate –fold difference in mRNA levels (DESeq mapped reads); red (tachyzoite), green (merozoite).

RNAs with ≥ 8-fold difference in abundance at each life cycle stage were parsed, and distributions into eleven gene product or functional categories are shown as pie charts (Figure 2B, Additional file 1: Table S1A, B). The global comparison identified hypothetical genes as the most abundant set of stage-specific genes (146/47% in merozoites versus 294/64% in tachyzoites) expressed between the two developmental stages examined. The RNA-Seq data will also serve to annotate, and thereby significantly reduce, the number of hypothetical genes found in both samples. Genes related to metabolism or the cytoskeleton were not differentially regulated, with only a few exceptions. In contrast, the majority of genes implicated in host parasite interactions, such as secreted and surface proteins, were regulated in a stage-specific fashion (Figure 3). For example, significantly more surface (SRS) proteins were expressed in merozoites than in tachyzoites (40 versus 12), and their expression was restricted exclusively to the merozoite developmental stage. In striking contrast, the majority of microneme-, rhoptry-, and dense granule-specific secreted proteins were not expressed in merozoites, suggesting the existence of an unannotated suite of MIC, ROP and GRA proteins that promote merozoite asexual replication.

### Merozoite specific gene families

Several large, unannotated gene families containing many predicted secreted and/or membrane-associated proteins, referred to as the *T. gondii* Family A-E proteins were identified to be largely merozoite stage-regulated (Additional file 2: Figure S1). *Family A* is predominantly merozoite-specific, with 29 of 33 members expressed above the ≥ 8-fold abundance threshold. Only one *Family A* gene was expressed exclusively in tachyzoites (Additional file 2: Figure S1A and Additional file 1: Table S2). Members of the remaining four families (with the exception of *Family E*) were likewise strongly expressed in merozoites (>4-fold change to tachyzoites), but just below the threshold (Additional file 2: Figure S1B). The “miscellaneous” category (Figure 2) also contains members of additional structural protein families many of whose members are strongly stage-regulated. One example is the Lysine-Arginine Rich Unidentified Function (KRUF) family of proteins [10] (Additional file 1: Table S3). Twelve of the 14 KRUF family members are strongly expressed in tachyzoites but not expressed (≥8-fold lower) in merozoites.

### Genome annotation

RNA-Seq data provides valuable information about the accuracy of existing gene models in ToxoDB [11]. In this case the newly generated datasets complement existing RNA-Seq data, currently from tachyzoite, bradyzoite, and oocyst stages. To estimate how many gene models have no associated RNA-Seq data we used ToxoDB search strategies. While a detailed revision of ToxoDB gene models based on RNA-Seq data will have to await additional data from cat-derived gametocytes, we used simple ToxoDB in-silico search strategies with stringent criteria to identify merozoite-specific gene models that are currently without any evidence for expression. Several examples could be discovered by collating all predicted gene models for which no or insufficient (<40^th^ percentile) evidence for RNA (n = 42) or protein expression (n = 38) exist at any stage of development and comparing them with the datasets developed in this study. We eliminated any entries with RPKM values >10 in tachyzoites and <10 in merozoites resulting in 10 genes for which significant expression is only documented in merozoites. The group comprises 6 *Family A* genes (Additional file 2: Figure S1), three hypothetical genes and *SRS15B*. Although such search strategies are difficult to threshold and require manual follow-up and verification, they represent examples of data mining efforts which are well within reach for experienced ToxoDB users. In addition, fortuitous discovery of large numbers of merozoite-specific RNA-Seq reads mapping to regions without predicted gene models, e.g. in a 4 kb region between TGME49_297820 and TGME49_297830 on chromosome II, indicates that systematic mapping of merozoite data will lead to discovery and annotation of many novel protein coding genes.

### Metabolic capacity is similar in merozoites and tachyzoites

Merozoite populations expand rapidly by asexual division whilst remaining strictly confined to the enterocyte monolayer (Figure 1A, B) and presumably are confronted with less variability in terms of bioavailability of metabolites and building blocks than tachyzoites which infect different cell types. In addition, the parasite is ideally positioned to intercept the stream of nutrients flowing from the apical brush border to the basolateral face of the host enterocyte. In the absence of data on enzyme activity levels, mRNA expression can be used effectively to model metabolic flux distributions and stage-specific changes [12,13]. Global comparative analysis of metabolic gene expression was done on the subset of genes used for constructing the iCS382 metabolic model for *Toxoplasma* [14]. Although we identified 11 “tachyzoite-specific” and 7 “merozoite-specific” genes based on >8-fold difference in mRNA expression within this subset (Additional file 2: Figure S2), there is little evidence in our data that any of these were clustered either on chromosomes or in any particular metabolic pathway. Nevertheless, several genes of the energy metabolism are strongly regulated, with the glycolytic enzyme enolase 1 (*ENO1*, TGME49_268860) showing the highest difference in expression, albeit with relatively low RPKM values, and is effectively silenced in merozoites (RPKM 80 in tachyzoites, vs 1 in merozoites, adjusted p value: 1.74e-7). *ENO1* is a bradyzoite marker and thus not well expressed in tachyzoites to begin with. Indeed, *ENO1* appears to be dispensable in tachyzoites: cells with a targeted deletion of the gene grow well in vitro and in vivo, but cyst numbers in chronically infected mice are significantly decreased [15]. Conversely, the *ENO2* (a canonical tachyzoite marker) gene is strongly downregulated in bradyzoites. In merozoites *ENO2* mRNA is detected at ~2-fold higher levels (RPKM 620) than in tachyzoites, which points to an adaptive response geared towards a higher glycolytic throughput compared to tachyzoites. Although a more detailed analysis of energy metabolism would require RNA-Seq data for these enzymes in all coccidial stages and also bradyzoites integrated into a flux model such as iCS382 [14], it is tempting to speculate that these adaptations in merozoites help sustain rapid growth in conditions of low oxygen tension in the gut. In addition to their function in glycolysis, nuclear targeted ENO isoenzymes were shown bind to chromosomal DNA and modulate gene expression [15]. The significance of low levels of ENO1 expression in the tachyzoite dataset and the tight silencing of the gene in merozoites in the context of their regulatory functions remain to be investigated. The stage-specific regulation of *ENO* expression is also consistent with 3.5 fold higher levels of phosphoglycerate kinase *PGKI* (TGME49_318230) RNA-Seq reads in merozoites. In line with this, differential expression of the two glycolytic lactate dehydrogenase isoenzymes LDH1 and LDH2 [16] in merozoites is very similar to tachyzoites but even more pronounced: virtual silencing of LDH2 (RPKM 2) but strong expression of LDH1 (RPKM 962 vs 472 in tachyzoites). There are also several examples for tachyzoite-specific expression of metabolic factors: both NADPH-generating glucose-6-phosphate 1-dehydrogenase (TGME49_294200), an enzyme of the pentose phosphate pathway, and 8-amino-7-oxononanoate synthase (TGME49_290970), a key enzyme in the biotin synthesis pathway, are not expressed in merozoites (RPKMs 5 and 1, respectively). Biotin provides the prosthetic group for carboxyl transferases and has essential functions in many anabolic and catabolic reactions. Because biotin is contained in food but also supplied by gut microorganisms, a complete shutdown of its synthesis in merozoites suggests that developmental stages in enterocytes rely entirely on scavenging the easily accessible protein.

The most strongly regulated metabolic gene in merozoites relative to tachyzoites is glycerol-3-phosphate dehydrogenase (NAD^+^) (TGME49_210260), with essentially no expression in the latter (RPKM 12) but a 20-fold higher expression in merozoites (adjusted p-value: 2.7e-21) (Additional file 2: Figure S2). RNA-Seq reads for the homoserine O-acetyltransferase gene coding for an enzyme of the methionine biosynthesis pathway are similarly increased in merozoites. Interestingly, expression of two genes involved in purine metabolism, adenylosuccinate synthetase, (a.k.a., *IMP-aspartate ligase*, TGME49_279450), and hypoxanthine-xanthine-guanine phosphoribosyl transferase (*HXGPRT*, TGME49_200320) is > 8-fold higher in merozoites. The latter, a key enzyme in the purine salvage pathway [17], is expressed at a very high level in merozoites with an RPKM value of 882 compared to 106 in tachyzoites. Another purine metabolism gene, purine nucleoside phosphorylase (*PNP*, TGME49_307030), is expressed 6-fold higher in merozoites (RPKM 88, adjusted p-value: 1.9e-2), suggesting a possible expansion of purine salvage throughput at least during the asexual phase of parasite population growth during enteroepithelial development.

Altogether, we conclude that in contrast to the marked differences in gene expression of secreted proteins in tachyzoites and merozoites, the expression of genes involved in metabolic processes is largely unaffected. Hence, metabolism is predicted to be broadly similar between tachyzoites and merozoites, although there may be some fine-tuning in the latter for optimal growth in enterocytes. However, no fundamental differences were detectable that would indicate a radically different environment or nutrient availability for growth in enterocytes. A more detailed examination and testing of specific hypotheses using for example the recently developed flux balance analysis models to identify stage-specific metabolic bottlenecks [14] will be required to confirm or refute this current interpretation as well as identify potential drug targets.

### SRS proteins are widely expressed in merozoites

The *T. gondii* genome encodes several distinct, coccidian-specific surface gene families, including the *SRS* and SAG-unrelated surface antigens (*SUSA*) [18]. Arguably the most strikingly regulated set of genes in the merozoite dataset were those coding for the 111 members of the SRS superfamily of proteins annotated in ToxoDB (Version 8.1); expression of more than 52 members of this family was present in merozoites, whereas a separate set of 14 *SRS* genes were expressed exclusively in tachyzoites (Figure 3). Nearly half of the merozoite expressed genes coding for SRS proteins were present in 5 clusters, *SRS12, 15, 22, 26* and *55*. SRS proteins are involved in attachment to host cells, but also provoke immune reactions and regulate parasite virulence, which is thought to promote the formation of tissue cysts in intermediate hosts in order to establish persistent, latent infections that facilitate transmission of infection to the definitive host. Previous work has showed that tachyzoites differentially express a number of *SRS* genes [18], and these expression differences have been postulated to account for the ability of this stage to invade a broader range of host cells than other coccidians [10]. However, merozoites, which only infect a single cell type (the feline enterocyte) co-dominantly expressed a large repertoire of 52 SRS proteins in a developmental life-cycle stage-dependent manner (Additional file 2: Figure S3). Alternatively, recent work has established that the SRS fold is present in the 10 member Pfs-230-related 6-Cys family of *Plasmodium* adhesins [19] that facilitate gamete-gamete recognition and promote gamete recognition and fertilization. This may suggest that the merozoite-restricted *SRS* genes are less relevant for attachment and invasion within enterocytes, but rather promote gamete development and fertilization. Given the demonstrated ability of SRS proteins to promote immune responses, it is also conceivable that they play a role in stimulating intestinal inflammation and diarrhea, to facilitate the production and dispersal of oocysts.

**Figure 4:**
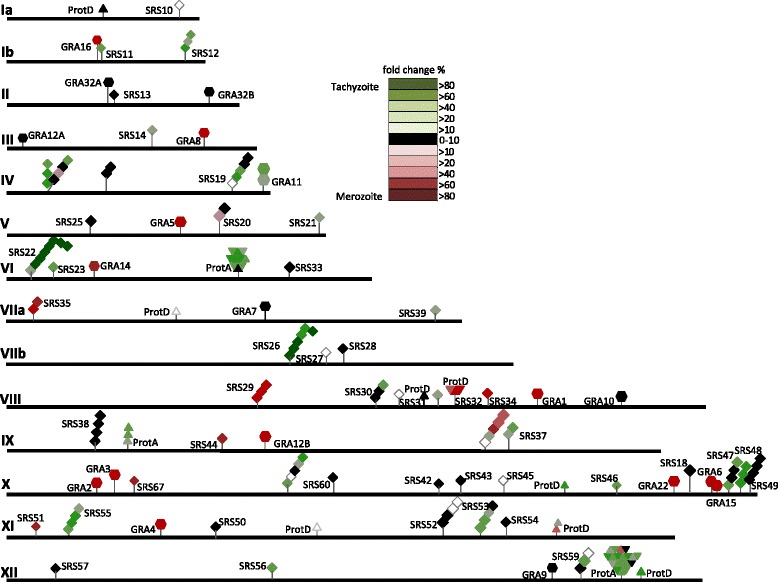
**Chromosomal distribution and relative expression levels of SRS, GRA, and** ***T. gondii*** **protein A and D families.** The chromosomal position and distribution of *SRS* (◊), *GRA* (⎔) and *T. gondii protein A* and *D* families (Δ) that are differentially expressed between merozoites and tachyzoites is displayed. Tandemly repeated genes are shown as clusters. Uncoloured genes were not expressed in either tachyzoite or merozoite >stage (RPKM <10). Black coloured genes were not differentially expressed between tachyzoites and merozoites. The shade of red (induced in tachyzoites relative to merozoites) or green (induced in merozoites relative to tachyzoites) indicated the fold increase in expression relative to the other life cycle stage. The chromosomal position of *SRS* pseudogenes is not displayed. The majority of *GRA* genes were upregulated in tachyzoites. Only *GRA11* gene expression was specifically induced in merozoites. The majority of *SRS*s were upregulated in merozoites. 52 *SRS* genes were upregulated in merozoites whereas only 14 were upregulated in tachyzoites. Genes in each cluster tended to be coordinately regulated according to life cycle stage, with only 4 exceptions: One gene in each of the *SRS16*, *SRS36*, *T. gondii Family A* (Chromosome XII) and *T. gondii Family D* (Chromosome XI) clusters was upregulated in tachyzoites, relative to merozoites.

The striking stage-specificity of expression of *SRS* genes in merozoites versus tachyzoites (and as previously observed for merozoites versus bradyzoites) raises questions about how their expression is regulated. One clue to this can be obtained from analysis of chromosomal distribution of the various *SRS* genes [10,18]. However, we find little evidence of concerted clustering of merozoite- or tachyzoite-specific *SRS* genes on distinct chromosomes (Figure 4), which is not a complete surprise since *T. gondii SRS* genes are dispersed across all chromosomes and not exclusively in a subtelomeric regional distribution pattern for surface antigen genes that is characteristic for other protozoa [20-23]. Thus, rather than relying on sequential expression of a single gene or promoting rapid ectopic recombination rates to generate surface antigenic variation, *T. gondii* expresses relatively large sets of non-overlapping, co-dominant *SRS* transcripts [18,24].

### Merozoites express a distinct set of microneme proteins

**Figure 5:**
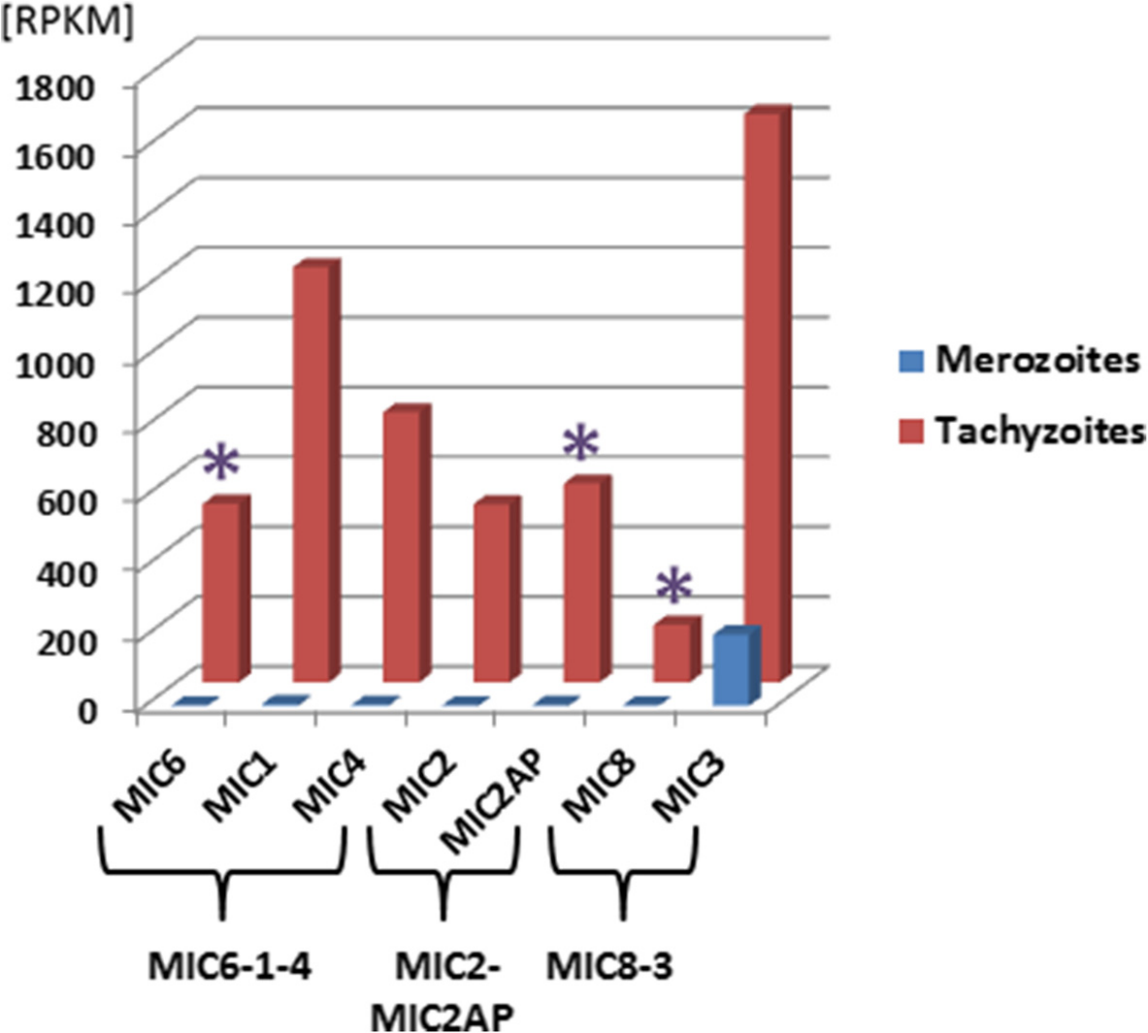
**Differential expression of three MIC escorter/adhesin complexes.** Expression values are indicated as [RPKM], asterisks: genes coding for MIC escorter proteins.

Micronemes are small organelles clustered at the anterior end of apicomplexan parasites and generally secrete adhesive proteins that are important for motility and invasion. Microneme (MIC) proteins contain one or several adhesive domains such as thrombospondin, von Willebrand factor A, epidermal growth factor (EGF) or PAN-domains (reviewed in [25]). Comparative analysis of RNA-Seq data shows that the majority of the 26 *Toxoplasma* MICs and MIC2 associated protein (MIC2AP) accumulate a high number of mapped tachyzoite reads (Additional file 2: Figure S4). Conversely, the range of *MIC* genes that are specifically expressed in merozoites is considerably reduced and restricted to the highly expressed genes coding for PAN-domain containing proteins MIC17A-C (Additional file 2: Figures S4 and S5), *MIC12*, a gene annotated as a putative MIC, and a microneme-like protein. Interestingly, both MIC2 and MIC2AP [26], as well as MIC6-1-4 [27], which are strongly expressed in tachyzoites and secreted as functional complexes, are virtually silenced in merozoites (Figure 5). Similarly, the RNA-Seq reads for the *MIC3* escorter *MIC8* [28] are >100-fold lower in merozoites, whilst *MIC3* mRNAs are only 6-fold less abundant. Since trafficking to the organelles, as well as deployment on the surface, seems to occur in complexes as a rule [29], we analyzed the predicted topologies of the six merozoite-specific MIC proteins. None had the predicted transmembrane domain and cytoplasmic tail typical for canonical escorters such as MIC6 [30]. In tachyzoites these adhesin complexes are essential components of the molecular motor and play a key role in providing the machinery for gliding motility [31].

The number of microneme organelles of different Apicomplexa has been correlated with the requirement for gliding motility [25], ranging from many in *Eimeria* sporozoites and merozoites [32] to none in *Theileria* zoites [33]. *Toxoplasma* tachyzoites, which display vigorous gliding motility and are able to cross cell barriers, have many microneme organelles and express a wide range of MIC proteins. Although *T. gondii* merozoites have a high number of microneme organelles [5], the complement of expressed *MIC* and other genes (e.g. *AMA1* [34]) coding for proteins secreted from micronemes appears to be significantly restricted, suggesting that the size of the expressed protein complement is not necessarily correlated to the number of organelles present in the cell. On the other hand, the finding that prominent tachyzoite MIC complexes are not expressed in merozoites (Figures 3 and 5), could be explained by fundamental differences in their mode of gliding motility and cell invasion in contrast to the extraintestinal developmental stages. Because merozoite proliferation is restricted to enterocytes, the most obvious difference is that mature merozoites egress into the gut lumen and invade new host enterocytes from the exposed apical side. This requires some migration through intestinal contents and within the mucus layer rather than through intercellular spaces and on cell surfaces. Because freshly isolated extracellular merozoites display vigorous gliding motility (data not shown) it is unlikely that these stages are without surface adhesins linked to the glideosome complex [31,35]. Rather, as yet unknown MIC adhesins specific for enterocyte surface receptors and containing non-canonical adhesive domains will likely be discovered among hypothetical proteins that are expressed in a merozoite stage-specific manner.

### Merozoites only weakly express genes of the tachyzoite moving junction complex

**Figure 6:**
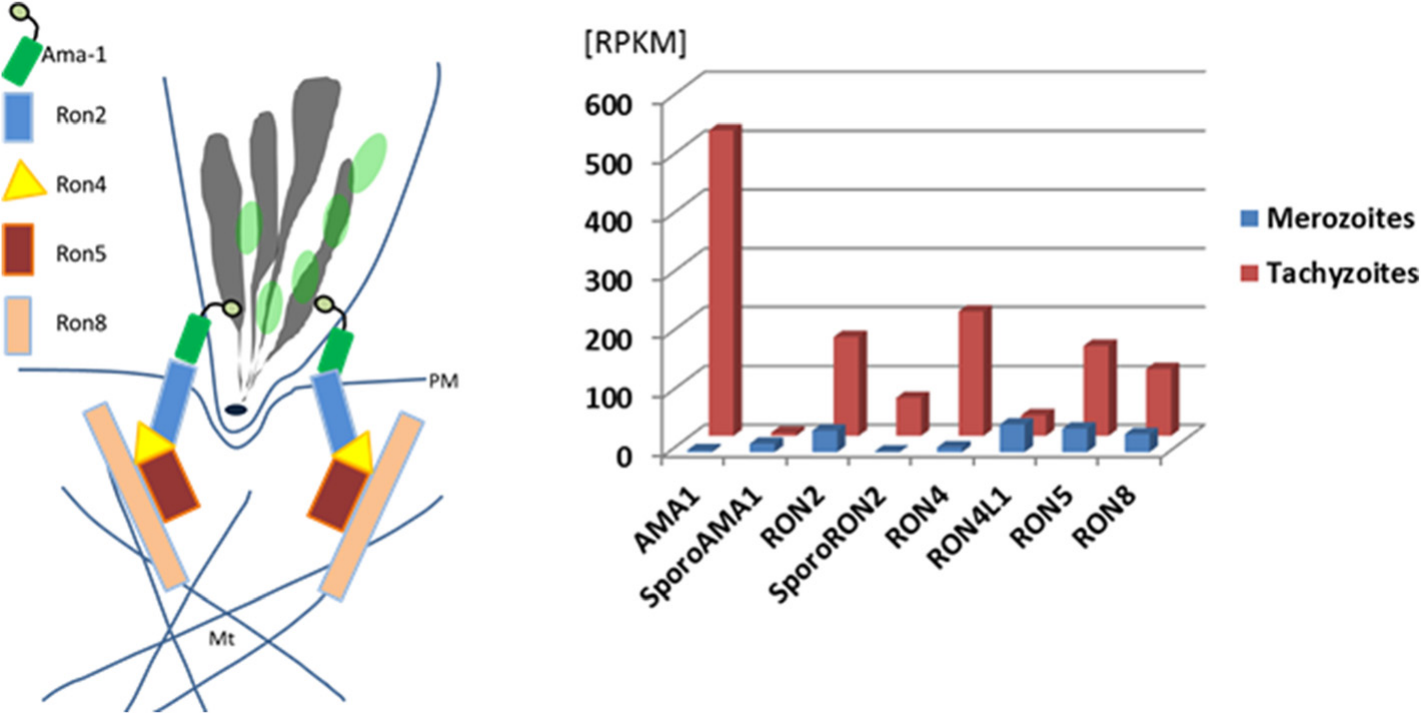
**Genes coding for components the tachyzoite moving junction invasion machinery are expressed at significantly lower levels in merozoites.** Cartoon depicting the RON and AMA protein assembly at the moving junction complex (left); bar graph showing expression (RPKM values) of components and paralogs in tachyzoites and merozoites. Mt, microtubules; PM, host cell plasma membrane.

There is a striking difference in expression of genes coding for rhoptry neck proteins (RONs) and AMA1 in tachyzoites versus merozoites (Figure 6). In particular, mRNA levels of genes coding for proteins that combine to form the moving junction as part of the tachyzoite invasion machinery (i.e.*,* AMA1, RON2, RON4, RON 5 and RON8; Figure 6) [36,37] are dramatically lower in merozoites. This supports the contention, raised above, that the mechanism of gliding motility and by extension cell invasion is distinct in merozoites. The dramatically lower expression of the canonical tachyzoite AMA1 (TGME49_255260) in merozoites is not compensated by a significant increase in expression of the sporozoite-specific AMA1 paralog and the associated sporoRON2 [38] is not expressed. A third *Toxoplasma* AMA1 paralog (TGME49_300130) is weakly expressed in both stages, and its functional role in invasion, if any, remains to be demonstrated. Interestingly, previous claims that AMA1 is essential for tachyzoite invasion [39] has been revised in light of recent data showing that AMA1 knockout tachyzoites invade host cells normally *in vitro* [40]. On the one hand, gene expression data alone predicts that invasion via the massively expanded surface of the enterocyte brush border by merozoites is distinct from the well described process observed in extraintestinal stages. On the other, the exact requirements and concepts for formation of a tight junction [36,41-44] which will be moved towards the posterior end of the invading parasite by motor proteins are currently being revisited [40,45]. While gene expression data indicates fundamental differences in cell invasion by merozoites, the question whether merozoites assemble an alternative moving junction machinery for invasion or invade enterocytes by an entirely different mechanism, e.g. based on membrane-zippering such as in *Theileria* [46] or *Neospora* [47], requires further investigation.

### Merozoites express a distinct set of rhoptry proteins

We also investigated RNA expression for a group of 57 genes coding for rhoptry proteins (ROPs) compiled from annotated gene models on ToxoDB (Version 8.1) [48] and expanded by manual annotation (Additional file 1: Table S4). In total 24 *ROP* genes were significantly differentially expressed (≥8-fold abundance threshold) between tachyzoites and merozoites (Figure 3 and Additional file 2: Figure S6); 19 genes were considered not expressed in merozoites (RPKM values were <10 in all cases) whereas two were expressed at very low levels (RPKM < 40) in merozoites. Four *ROP* genes were, however, expressed specifically in merozoites. Interestingly, in a survey of known functions of ROP proteins, we find that the prominent murine virulence factors ROP16 and ROP18/5 are not expressed in merozoites.

ROP16 and 18, which both possess a conserved catalytic triad and phosphoryltransfer activity, have been identified as secreted, strain-specific virulence factors in mice. ROP16 phosphorylates the signal transducers and activators of transcription (STATs) 3 and 6, and regulates interleukin 12 levels [49,50]. The polymorphic ROP18 kinase phosphorylates and thus inhibits loading of immunity-related GTPase (IRG) 6 onto the PV membrane, thereby preventing parasite clearance in infected mice by activated macrophages [51,52]. ROP5, a pseudokinase with an altered catalytic triad, regulates the kinase activity of ROP18 [53] possibly by an allosteric mechanism. Therefore, the absence of IRGs in cats is the simplest explanation for the silencing of the *ROP18* (and possibly the *ROP5*) gene in merozoites rendering expression and secretion of these ROPs as part of a defense strategy redundant during enteroepithelial development.

### Merozoites express a distinct set of dense granule proteins

We detected significant differences in expression within the current set of 25 annotated genes encoding dense granule (GRA) proteins (Figure 3, Additional file 2: Figure S7). There was a significant overall bias towards high expression in tachyzoites. Twelve *GRA* genes were not expressed in merozoites (RPKM < 10), whilst only 2 *GRA* genes (*GRA11* isoforms TGME49_212410 and TGME49_237800, which have, as yet, undefined functions) are >8-fold higher expressed in cat stages (18- and 13-fold, respectively; Figure 3). The high expression of *GRA22* in tachyzoites is intriguing, since this protein regulates parasite egress from host cells [54] and the absence of *GRA22* expression in merozoites is therefore puzzling. This might imply that egress from cat enterocytes is less coordinated by the parasite and this may partially explain why parasite development is asynchronous in the cat intestine. GRA genes expressed in tachyzoites fall into two main groups: moderately highly expressed *GRA* genes (RPKM values in the range of 100) code for proteins that are exported across the PV membrane that function to alter host gene expression (i.e*., GRA* 15, 16, and 24); versus highly expressed *GRA* genes (RPKMs > 10^3^) some of whose products participate in the formation of the tubulovesicular network (TVN) within the PV space. Our RNASeq data shows that GRA15 is not expressed in merozoites. This tachyzoite virulence factor has a well-defined role in the activation of several host cell pathways that trigger potent pro-inflammatory responses causally associated with tachyzoite to bradyzoite conversion [49,55-59]. In mice, parasite survival is dependent on combined expression of GRA15 with the STAT-activating form of ROP16 [49]. Both *GRA15* and *ROP16* genes are not expressed in merozoites, strongly suggesting that JAK-STAT3/6 and NF-κB signaling pathways in enterocytes of the definitive host are not targeted by these secreted factors. *GRA24* and *16* are also not expressed in merozoites. GRA24 was recently identified as a parasite soluble effector that traffics to the host cell nucleus, interacts with p38, and is thought to synergize with GRA15 to promote production of the pro-inflammatory cytokine, IL12 [60]. Similarly, GRA16 is exported to the host cell nucleus and affects transcription of host cell genes involved in metabolism and cell cycle processes [61]. Whether these host cellular processes are not targeted by merozoite gene products during their asexual expansion in cat enterocytes, or if as yet unannotated merozoite-specific paralogs exist to perform this function, remain to be established.

The RPKM values for *GRA2*, *4*, and *6* genes, coding for three proteins which are directly associated with the TVN in the PV [62,63], are >8-fold lower in merozoites compared to tachyzoites (Additional file 2: Figure S7). The silencing of these *GRA*s (RPKMs <10 and log [2]-fold change ≥ 8) in merozoites is consistent with electron microscopy data [5] showing that type C-E schizonts in cat enterocytes do not elaborate the extensive TVN which is typical of vacuolar spaces of tachyzoites, bradyzoites and type A and B schizonts. This suggests that later stage schizonts dispense with the TVN because of their different mode of cell division [5]; parasites in type C-E schizonts multiply by endopolygeny rather than by endodyogeny as early stage merozoites, tachyzoites and bradyzoites. Magno *et al*. [64] suggested that an important structural function of the TVN might be organization of daughter cells within the PV during multiplication. Involution of the TVN and repression of genes coding for factors associated with these membranes in merozoites is consistent with endopolygeny and attachment of parasites to residual bodies [5]. Conversely, the *GRA7* gene, whose 36 kDa product is involved in nutrient acquisition by trapping host endosomal-lysosomal vesicles in the PV [65], is expressed at ~4-fold lower levels in merozoites (Additional file 2: Figure S7) but is still relatively abundant with an RPKM value of 351 (1837 in tachyzoites). Thus, although not always associated with the PV membrane, GRA7 appears to be universally expressed throughout the life cycle [66].

Additional factors secreted by dense granules include Kazal-type protease inhibitors PI1, PI2 (TgME49_217430, TgME49_208450) [67-69] (Table S5), which are secreted into the PV and appear to be differentially regulated, despite strong conservation on the structural and sequence level. Whilst read numbers for the *PI1* gene, the most abundantly expressed protease inhibitor in tachyzoites [69], are >2-fold higher in merozoites, *PI2* is not expressed in merozoites (RPKMs 50-fold lower). Three of five additional genes annotated as Kazal-type family protease inhibitors (Table S5) show significantly higher expression in merozoites. However, whether the four predicted secreted members of this group also traffic via dense granules is unknown.

### *Trans* and *cis* regulators of merozoite and tachyzoite biology

Both *trans* and *cis* regulators are known to play crucial roles in the transcription of stage specific genes in *T. gondii*. The *trans*-acting ApiAP2 transcription factors were discovered in the Apicomplexa almost 10 years ago [70] and regulate various developmental processes in *T. gondii*, including bradyzoite conversion, with TgAP2XI-4 [71], and TgAP2IX-9 [72] promoting and restricting tissue cyst formation, respectively, as well as the tachyzoite cell cycle, with TgAP2XI-5 potentially regulating hundreds of cell cycle-dependent genes [73]. Using stringent criteria we identified three significantly merozoite- or tachyzoite-specifically expressed ApiAP2-coding genes in our study (Additional file 2: Figure S8). One merozoite-specific gene *TgAP2VIIa-I*, and two tachyzoite-specific genes, *TgAP2XII-I* and *TgAP2VIIa-II* showed >8-fold difference in their expression levels, suggesting that these transcription factors may play a role in regulating a suite of stage-specific genes. Relaxing the criterion to include genes differing ≥4-fold in mRNA abundance identified 8 additional tachyzoite-, and 2 additional merozoite-specific genes (a total of 13 regulated stage-specific TgAP2s). Interestingly, several other *TgAP2* genes were expressed at very low levels, and in the case of *TgAP2XII-3,* can be considered silenced in both stages. Overall, differential expression of *TgAP2* is not extensive between tachyzoite and merozoites, and this may reflect their similar life cycle fate as rapidly amplifying asexual stages. However, the evenly graded distribution of RPKM values (Additional file 2: Figure S8B) suggests that some differences may become more pronounced when merozoites differentiate to gametocytes. Moreover, the two datasets compared here do not represent synchronized populations, which would average out any dynamics occurring during the merogony cell cycle specifically during development from stage A through stage E schizonts. By contrast, 24 of the 67 predicted *TgAP2*-coding genes were found to be differentially expressed at different check-points of the tachyzoite cell cycle in a microarray analysis of synchronized parasites [74]. In addition, ApiAP2s have been shown to bind other transcriptional regulators such as histone lysine acetyltransferases [75], histone deacetlyase [76] and even other ApiAP2s [77], producing interactions that may confound correlations between transcription factor activities and their cognate gene transcript levels.

**Figure 7:**
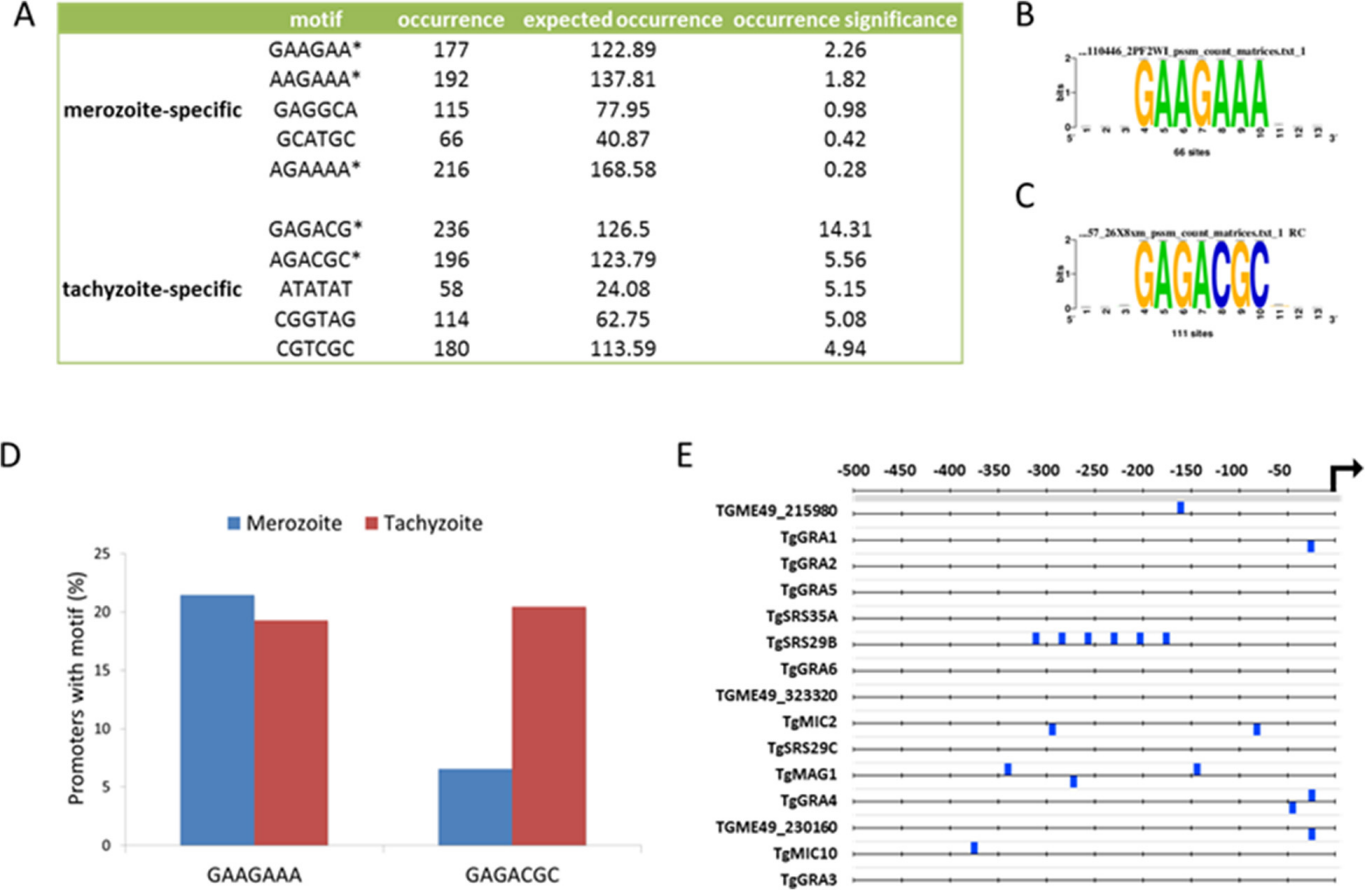
**Identification of** ***cis*** **regulatory elements in the predicted promoters of merozoite- and tachyzoite-specific genes. (A)** Enriched six-base motifs were identified within the predicted promoters of merozoite- and tachyzoite-specific genes. Listed for both stages are the five most significantly enriched motifs, their occurrence, expected occurrence, and occurrence significance. Asterisks indicate motifs overlapping with the putative *cis* regulatory elements for promoters of merozoite- and tachyzoite-specific genes, shown in **(B)** and **(C)**, respectively. **(D)** Percentage of promoters from merozoite- or tachyzoite-specific genes that contain either the GAAGAAA or GAGACGC putative *cis* elements. **(E)** Distribution of the GAGACGC *cis* element (blue) within the promoters of the fifteen most tachyzoite-specific genes. The predicted promoters correspond to the 500 bp genomic region directly upstream of the transcriptional start site, indicated by an arrow.

Next, we used the Regulatory Sequence Analysis Tools on-line computational resource (http://www.rsat.eu/) to perform a global analysis of upstream flanking sequences of stage-specifically expressed genes. The analysis returned several enriched six-base DNA motifs within the predicted promoters, i.e. 500 bp genomic regions upstream of the transcriptional start site of either merozoite- or tachyzoite-specific genes. Listed in Figure 7A are the five most significantly enriched 6 bp motifs found. Pattern matching between these enriched motifs produced an amalgamated seven-base DNA motif in both merozoite- and tachyzoite-specific gene promoters, representing putative *cis*-regulatory elements (Figure 7B and C, respectively). The GAAGAAA motif, present in 21.5% of merozoite promoters, was also present in 19.2% of tachyzoite promoters, indicating that this motif is unlikely to represent a merozoite-specific *cis*-regulatory element (Figure 7D). However, the GAGACGC motif is clearly enriched in the promoters of tachyzoite-specific genes (present in 20.4% of promoters) over merozoite-specific genes (6.5%). The distribution of this putative *cis*-regulatory element in the predicted promoters of the 15 most tachyzoite-specific genes is illustrated in Figure 7E. This motif is nearly identical to a motif (A/TGAGACG) previously identified as a functional *cis*-element in the promoters of dense granule genes, such as *TgGRA1*, *TgGRA2*, *TgGRA5* and *TgGRA6*, as well as the tachyzoite marker, *TgSRS29B* (aka *TgSAG1*) [78]. A sequence-specific transcription factor capable of activating gene transcription via this *cis* element has yet to be identified; however, it may play a significant role in promoting and/or maintaining a tachyzoite developmental state.

## Conclusions

Taken together, our data support a correlation between promiscuity of invasion and dissemination of the *T. gondii* parasite in a variety of host cells and tissues. In addition, the data presented here underscore the phenomenon of non-overlapping stage-specific expression of gene sets throughout development (e.g. *SRS*, *ROP*, *GRA*, *MIC*) as previously proposed [79] within the *Toxoplasma* life cycle [18]. Hence, the differentiation of bradyzoites into merozoites, which occurs only in the feline intestine, entails the shut down or downregulation of suites of tachyzoite-specific genes that would normally be activated to support *Toxoplama’s* promiscuous ability to invade and reproduce asexually in virtually any nucleated cell from essentially any warm-blooded animal. This study provides compelling datasets to begin to explore the suites of genes activated to support merozoite invasion, differentiation, and expansion in cat enterocytes and establishes *T. gondii* as an excellent genetically-amenable model system to study schizont asexual amplification prior to gamete development, the hallmark of coccidian development in the definitive hosts these coccidian parasites infect. Our data suggest that gliding motility and the formation of a moving junction (or an alternative invasion apparatus) requires machinery that is specifically adapted to breaching the enterocyte surface barrier for every cycle of replication, egress and invasion. Studies of the SRS proteins exclusively expressed during coccidian development will determine if these proteins promote attachment and invasion of feline enterocytes, or whether they participate in gamete-gamete recognition to promote fertilization, analogous to the orthologous role performed by related 6-Cys family proteins during *Plasmodium* development [80]. Understanding the governance of gene expression and regulation within these families, e.g*. via* interactions with the TgAP2 family of transcriptional regulators, may prove key to dissecting the full panorama of pathways that regulate the definitive coccidian lifecycle and aid in the complete and accurate annotation of the *T. gondii* genome database.

## Methods

### Isolation of enteroepithelial parasite stages from a cat infected with bradyzoites from sheep

Oocysts of the CZ *T. gondii* isolate (type II) were originally isolated from the feces of a captive Siberian tiger (*Panthera tigris altaica*) at the Dvůr Králové Zoo (Czech Republic). The strain isolation was performed in 2005 by Dr. B. Koudela, and the strain was maintained by passages between mice and cats [81,82]. After obtaining the strain from Dr. Koudela the parasites were passed once through mice and cats to generate ~50 million CZ oocysts, which were used to infect four sheep (10^4^ oocysts per sheep). Equal amounts of brain tissue containing bradyzoites in tissue cysts from three chronically infected sheep was homogenized and fed to cats (55 g per cat). A semi-quantitative analysis of parasite burden in sheep brain tissue was done by magnetic capture real time PCR, to confirm that equal quantities of parasites were inoculated into each cat but no definitive count of cysts was performed. Pilot experiments had established that this protocol results in strong and reproducible infections of cats. All feces from experimentally infected cats were collected and oocysts were enumerated by microscopy every day. For isolation of enteroepithelial stages of *T. gondii* a cat that was experimentally infected with brain cysts from a sheep was euthanized after onset of patency (day 5 post infection). The small intestine was clamped, removed, and immediately cooled to 0°C. All subsequent procedures for parasite isolation and purification were performed in a cold room (4°C) and on ice. The small intestine (~70 cm) was carefully rinsed with PBS, divided into four sections of equal length, and opened. Enterocytes were selectively removed from villi by gentle scraping with a rubber policeman (Figure 1B). Tissue samples for histology were taken before and after removal of enterocytes. The cells containing numerous parasites were washed off with ice cold PBS and collected by centrifugation (10 min, 900 × *g*, 2°C). Enterocyte preparations were evaluated microscopically after fixation and staining to identify parasite stages. The two enterocyte samples collected from two different sections of the small intestine were washed with buffer containing Tween80 and syringe-passaged The samples appeared to consist exclusively of free merozoites as no other stages (i.e. gamonts) could be identified by microscopy (Figure 1C).

Histological and fluorescence microscopy examination of small intestinal tissue sections revealed numerous parasite stages that were readily identified as merozoites, schizonts (Figure 1A) and also occasional gamonts due to their very distinctive morphologies. However, the final preparation was clearly highly enriched for merozoites allowing us to focus on interrogating RNA expression datasets specifically for differences between asexually developing merozoites and tachyzoites. The enrichment for merozoites or, more precisely, the paucity of gametocytes in the two biological replicates used for RNA-Seq, was indeed borne out by a lack of known gametocyte markers (e.g. genes coding for flagellar or oocyst wall proteins) in the RNA-Seq datasets. This is in contrast with a preliminary gene expression analysis of stages isolated at or past peak patency (Ramakrishnan et al. unpublished). CZ strain tachyzoites were grown *in vitro*; confluent human foreskin fibroblast (HFF) cells were infected at an MOI (>5) and harvested from synchronized cultures when 1/3 of the vacuoles had spontaneously lysed.

### Ethics statement

Experiments involving animals were performed under the direct supervision of a veterinary specialist, and according to Swiss law and guidelines on Animal Welfare and the specific regulations of the Canton of Zurich. Permit number 108/2010 covers all animal experiments presented in this paper and was approved by the Veterinary Office and the Ethics Committee of the Canton of Zurich (Kantonales Veterinäramt Zürich, Zollstrasse 20, 8090 Zürich, Switzerland).

### RNA isolation and quality control

Infected tissue and parasite samples were resuspended in 600 μl buffer RLT (QIAGEN) containing 10 μl/ml β-mercaptoethanol. Cell suspensions were then passed through a QIAshredder (QIAGEN) column by centrifugation at ≥ 8000 g for 1 min. The RNA was then purified using the RNeasy Mini Kit (QIAGEN) according to the manufacturer’s protocol (including an on-column DNA digest), and eluted in RNase-free water. The quality of the RNA was analysed using the Agilent RNA 6000 Pico Kit (Agilent) and a Bioanalyzer 2100 (Agilent) (Figure 1D). RNA concentration was determined using a Qubit fluorometer (Invitrogen) together with the RNA assay (Invitrogen).

The genome-wide transcriptome library, was produced using the MicroPolyA Purist Kit (Ambion) and the SOLiD Total RNA-Seq kit (Applied Biosystems). Briefly, approximately 300-500 ng of mRNA were enriched starting from 15-20 μg of total RNA, using MicroPolyA Purist Kit (two rounds of purification to receive only minor ribosomal RNA contamination). The quality and the quantity of the extracted polyA RNA was assessed using a Bioanalyzer (Agilent) Picochip and a Qubit fluorometer (Invitrogen), respectively. The mRNA was then fragmented using RNase III. Ligation of the adaptor mix and reverse transcription were performed following the manufacturer’s protocol. cDNA libraries were selected for fragment sizes between 150 and 250 bp, amplified for 15-18 cycles of PCR using barcoded adaptor primers and purified using the PureLink PCR micro kit (Invitrogen). Library size and concentration were assessed using a Bioanalyzer (Agilent) and a Qubit fluorometer (Invitrogen), respectively.

The poly-A transcriptome libraries were used for emulsion PCR (e-PCR) using a concentration of 0.5 pM. The barcoded libraries were pooled before e-PCR and the resultant beads were loaded on a full SOLiD 5 slide (Applied Biosystems), according to manufacturer’s instructions. SOLiD ToP Sequencing chemistry was used to produce paired-end (50 bp + 35 bp) sequencing reads.

### Comparative transcriptomics of merozoites and tachyzoites

We generated RNA-Seq datasets from CZ strain enteroepithelial parasites isolated from mechanically stripped enterocytes and from tachyzoites cultured in human foreskin fibroblasts on a SOLiD5 platform (Applied Biosystems). Two biological samples were sequenced for each stage (two separate parasite and RNA preparations from different sections of the small intestine as well as two different tachyzoite cultures). The samples contained different proportions of parasite and host RNA (Figure 1D). Adjustments to account for these differences were: i) removal of all reads mapping to tRNAs, and ribosomal RNAs prior to DESeq analysis, and ii) normalization and weighting by using transcripts lengths to calculate RPKMs. The reads in colorspace were aligned to the *T. gondii* ME49 genome from ToxoDB v8.1 using SHRiMP2 mapper [83,84].The count table of reads fitting the gene coordinates have been processed by Bioconductor library DESeq [8,85] to produce the table that includes the mean read number for each gene together with a p-value and fold change. We have applied an 8-fold (log2 ≥ [3]) change as a cut-off for differential expression.

### Immunofluorescence assay and microscopy

Samples from the small intestine of infected cats were embedded in paraffin. Sections of 3 μm were placed on slides with a positively charged coat. After drying over night at 37°C, slides were deparaffinized 3 times 2 min in xylene and then washed twice 1 min in 100% ethanol. Slides were then washed 1 min each in 96%, 70% ethanol, and water. Slides were then submerged in alkaline Target Retrieval Solution pH 9 (Dako), boiled for 20 min in a pressure cooker at 96°C and transferred to water. Permeabilization was done in PBS/0.3% Triton X-100 (permeabilization buffer, PB) for 5 min. Blocking was performed in rabbit serum for at least 1 h at RT. After two washes in PB for 5 min, sheep immune serum against *T. gondii* diluted in 20% rabbit serum/PB was used to label the parasites by overnight incubation at 4°C. Slides were washed three times with PB for 5 min and samples were incubated with rabbit anti-sheep FITC in 20% rabbit serum/PB for 1 h at RT. Slides were then washed once in PB and the nuclei were counter stained with 1 μg/ml DAPI in PB for 15 min. Slides were washed again twice with PB for 5 min and the samples were mounted using Vectashield (Vector Laboratories). Imaging was performed using a Leica DMI 6000 B microscope and the Leica LAS AF software. Images were processed using ImageJ version 1.47.

### Availability of supporting data

The raw sequence data and a complete description of the study are available at the European Nucleotide Archive (Study accession no. PRJEB7935). The data are also available via Toxoplasma Genomics Resource, ToxoDB, [http://www.toxodb.org/toxo/]. All other data sets supporting the results of this article are included within the article and its additional files.

## Additional files

Additional file 1: Table S1A. Significantly higher expressed genes in merozoites (see also Figure 2). **Table S1B.** Significantly higher expressed genes in tachyzoites (see also Figure 2). **Table S2.** List of annotated *T. gondii Family A* genes. The color key for the expression heat map (log2 fold change) is included. **Table S3.** Differential expression of *KRUF* family genes. **Table S4.** List of definitively and provisionally annotated rhoptry (*ROP*) genes. The color key for the expression heat map (log2 fold change) is included. **Table S5.** Protease inhibitors including differentially expressed secreted Kazal-type proteins. The color key for the expression heat map (log2 fold change) is included. Additional file 2: Figure S1- S8. Differential expression of – T. gondii genes and gene families: S1. Differential expression of – T. gondii Family A-E genes. S2. Differential expression of metabolic genes. S3. Differential expression of SRS genes. S4. Differential expression of microneme (MIC) genes annotated in ToxoDB. S5. Differential expression of genes coding for PAN-domain containing proteins. S6. Differential expression of annotated rhoptry (ROP) genes. S7. Differential expression of annotated dense granule (GRA) genes. S8. Differential regulation of TgAP2 genes.
