# Supplementary material for: Asexual expansion of Toxoplasma gondii merozoites is distinct from tachyzoites and entails expression of non-overlapping gene families to attach, invade, and replicate within feline enterocytes
Source: BMC Genomics. 2015 Feb 13;16(1):66. doi: 10.1186/s12864-015-1225-x (PMC4340605; doi:10.1186/s12864-015-1225-x)
Supplement: Additional file 1: Table S1A. — Significantly higher expressed genes in merozoites (see also Figure 2). Table S1B. Significantly higher expressed genes in tachyzoites (see also Figure 2). Table S2. List of annotated T. gondii Family A genes. The color key for the expression heat map (log2 fold change) is included. Table S3. Differential expression of KRUF family genes. Table S4. List of definitively and provisionally annotated rhoptry (ROP) genes. The color key for the expression heat map (log2 fold change) is included. Table S5. Protease inhibitors including differentially expressed secreted Kazal-type proteins. The color key for the expression heat map (log2 fold change) is included. [file 12864_2015_1225_MOESM1_ESM.pdf]

Additional file 2: Table S1A

Significantly higher expressed genes in merozoites (see also Figure 2).

Descending order: gene IDs and log2-fold changes (DESeq values) are indicated.

Gene ID	fold-chng (log2)	Gene ID	fold-chng (log2)	Gene ID	fold-chng (log2)	Gene ID	fold-chng (log2)
TGME49_295662	-5.8	TGME49_278410	-4.6	TGME49_301470	-3.7	TGME49_286782	-3.0
TGME49_273980	-5.6	TGME49_243170	-4.6	TGME49_231060	-3.7	TGME49_205390	-3.0
TGME49_238480	-5.6	TGME49_210330	-4.6	TGME49_287250	-3.7	TGME49_320770	-3.0
TGME49_266650	-5.6	TGME49_320090	-4.6	TGME49_315240	-3.7	TGME49_241165	-3.0
TGME49_237080	-5.6	TGME49_291005	-4.6	TGME49_231110	-3.6	TGME49_309352	-3.0
TGME49_243150	-5.6	TGME49_278330	-4.6	TGME49_283460	-3.6	TGME49_203580	-3.0
TGME49_320240	-5.5	TGME49_213850	-4.5	TGME49_237810	-3.6	TGME49_216155	-3.0
TGME49_238520	-5.5	TGME49_320170	-4.5	TGME49_212420	-3.6	TGME49_278245	-3.0
TGME49_277572	-5.5	TGME49_291920	-4.5	TGME49_278300	-3.6	TGME49_315400	-3.0
TGME49_232400	-5.5	TGME49_221560	-4.5	TGME49_278400	-3.6	TGME49_278240	-3.0
TGME49_320250	-5.5	TGME49_283470	-4.5	TGME49_238440	-3.6	TGME49_315350	-3.0
TGME49_266600	-5.5	TGME49_315370	-4.5	TGME49_210320	-3.6	TGME49_280470	-3.0
TGME49_306050	-5.5	TGME49_209470	-4.5	TGME49_279450	-3.6	TGME49_293690	-3.0
TGME49_278360	-5.5	TGME49_266340	-4.5	TGME49_200480	-3.6	TGME49_269365	-3.0
TGME49_321800	-5.4	TGME49_231390	-4.5	TGME49_214420	-3.6	TGME49_230667	-3.0
TGME49_236975	-5.4	TGME49_267470	-4.5	TGME49_244180	-3.6		
TGME49_238500	-5.4	TGME49_234380	-4.4	TGME49_301480	-3.6		
TGME49_238850	-5.4	TGME49_314250	-4.4	TGME49_280510	-3.6		
TGME49_259410	-5.4	TGME49_277230	-4.4	TGME49_321270	-3.6		
TGME49_238460	-5.4	TGME49_319090	-4.4	TGME49_286150	-3.5		
TGME49_296640	-5.4	TGME49_200250	-4.4	TGME49_276980	-3.5		
TGME49_238530	-5.4	TGME49_243378	-4.4	TGME49_278100	-3.5		
TGME49_202060	-5.3	TGME49_224905	-4.4	TGME49_247560	-3.5		
TGME49_238490	-5.3	TGME49_315390	-4.4	TGME49_250920	-3.5		
TGME49_278320	-5.3	TGME49_211280	-4.4	TGME49_266330	-3.5		
TGME49_259290	-5.3	TGME49_279000	-4.4	TGME49_283495	-3.5		
TGME49_291830	-5.3	TGME49_268270	-4.3	TGME49_269110	-3.5		
TGME49_329700	-5.3	TGME49_223830	-4.3	TGME49_261530	-3.5		
TGME49_259300	-5.3	TGME49_201180	-4.3	TGME49_268930	-3.5		
TGME49_202050	-5.3	TGME49_266335	-4.3	TGME49_263350	-3.5		
TGME49_238470	-5.3	TGME49_202930	-4.3	TGME49_327300	-3.5		
TGME49_207005	-5.3	TGME49_219828	-4.3	TGME49_229270	-3.4		
TGME49_207010	-5.2	TGME49_306270	-4.3	TGME49_246110	-3.4		
TGME49_321490	-5.2	TGME49_268800	-4.3	TGME49_238915	-3.4		
TGME49_307640	-5.2	TGME49_208850	-4.3	TGME49_316890	-3.4		
TGME49_243140	-5.2	TGME49_210260	-4.3	TGME49_271980	-3.4		
TGME49_243180	-5.2	TGME49_293372	-4.3	TGME49_233370	-3.4		
TGME49_260460	-5.2	TGME49_214190	-4.3	TGME49_228420	-3.4		
TGME49_278340	-5.2	TGME49_273110	-4.2	TGME49_200320	-3.4		
TGME49_251910	-5.2	TGME49_206620	-4.2	TGME49_300080	-3.4		
TGME49_319630	-5.2	TGME49_223030	-4.2	TGME49_236290	-3.4		
TGME49_243130	-5.2	TGME49_246070	-4.2	TGME49_259890	-3.4		
TGME49_287040	-5.2	TGME49_213430	-4.2	TGME49_316670	-3.4		
TGME49_202065	-5.2	TGME49_237070	-4.2	TGME49_235000	-3.4		
TGME49_254760	-5.2	TGME49_275660	-4.2	TGME49_265320	-3.3		
TGME49_278420	-5.1	TGME49_265030	-4.2	TGME49_233360	-3.3		
TGME49_243940	-5.1	TGME49_311700	-4.2	TGME49_306455	-3.3		
TGME49_251900	-5.1	TGME49_212410	-4.2	TGME49_311300	-3.3		
TGME49_245600	-5.1	TGME49_264630	-4.2	TGME49_212937	-3.3		
TGME49_207015	-5.1	TGME49_210740	-4.2	TGME49_214070	-3.3		
TGME49_255460	-5.1	TGME49_222188	-4.1	TGME49_248425	-3.3		
TGME49_205210	-5.0	TGME49_275380	-4.1	TGME49_205480	-3.3		
TGME49_267680	-5.0	TGME49_275355	-4.1	TGME49_217380	-3.3		
TGME49_321280	-5.0	TGME49_309300	-4.1	TGME49_248470	-3.3		
TGME49_305110	-5.0	TGME49_200270	-4.1	TGME49_307090	-3.3		
TGME49_229320	-5.0	TGME49_278090	-4.1	TGME49_247660	-3.3		
TGME49_231880	-5.0	TGME49_200240	-4.1	TGME49_258840	-3.3		
TGME49_224750	-5.0	TGME49_217370	-4.1	TGME49_254060	-3.3		
TGME49_219742	-5.0	TGME49_257572	-4.0	TGME49_316410	-3.3		
TGME49_320230	-5.0	TGME49_207965	-4.0	TGME49_256788	-3.3		
TGME49_243160	-5.0	TGME49_235010	-4.0	TGME49_281930	-3.3		
TGME49_320560	-5.0	TGME49_217720	-4.0	TGME49_228310	-3.3		
TGME49_259270	-5.0	TGME49_289370	-4.0	TGME49_244900	-3.3		
TGME49_278180	-5.0	TGME49_320730	-4.0	TGME49_255480	-3.3		
TGME49_226450	-4.9	TGME49_202110	-4.0	TGME49_258575	-3.3		
TGME49_274170	-4.9	TGME49_234940	-4.0	TGME49_209515	-3.3		
TGME49_222080	-4.9	TGME49_207730	-4.0	TGME49_204000	-3.2		
TGME49_224790	-4.9	TGME49_210095	-4.0	TGME49_226385	-3.2		
TGME49_329710	-4.8	TGME49_281350	-4.0	TGME49_203590	-3.2		
TGME49_278380	-4.8	TGME49_320290	-4.0	TGME49_298960	-3.2		
TGME49_243120	-4.8	TGME49_203500	-4.0	TGME49_266710	-3.2		
TGME49_321470	-4.8	TGME49_223550	-4.0	TGME49_237595	-3.2		
TGME49_266065	-4.8	TGME49_243110	-4.0	TGME49_321480	-3.2		
TGME49_281360	-4.8	TGME49_202100	-4.0	TGME49_316580	-3.2		
TGME49_291910	-4.8	TGME49_200230	-4.0	TGME49_318220	-3.2		
TGME49_267980	-4.8	TGME49_202090	-3.9	TGME49_270660	-3.2		
TGME49_278390	-4.8	TGME49_243382	-3.9	TGME49_309990	-3.2		
TGME49_286778	-4.8	TGME49_236940	-3.9	TGME49_271210	-3.2		
TGME49_273340	-4.8	TGME49_243190	-3.9	TGME49_219370	-3.2		
TGME49_312690	-4.8	TGME49_203340	-3.9	TGME49_248210	-3.2		
TGME49_278290	-4.8	TGME49_229230	-3.9	TGME49_249510	-3.2		
TGME49_219348	-4.7	TGME49_305220	-3.9	TGME49_252860	-3.1		
TGME49_254430	-4.7	TGME49_281970	-3.9	TGME49_295640	-3.1		
TGME49_239090	-4.7	TGME49_295995	-3.8	TGME49_244450	-3.1		
TGME49_234060	-4.7	TGME49_321710	-3.8	TGME49_224760	-3.1		
TGME49_235390	-4.7	TGME49_233330	-3.8	TGME49_207250	-3.1		
TGME49_316690	-4.7	TGME49_207790	-3.8	TGME49_310420	-3.1		
TGME49_201100	-4.7	TGME49_325200	-3.8	TGME49_292260	-3.1		
TGME49_278350	-4.7	TGME49_206660	-3.8	TGME49_207780	-3.1		
TGME49_278370	-4.7	TGME49_204870	-3.8	TGME49_217710	-3.1		
TGME49_278430	-4.7	TGME49_321700	-3.8	TGME49_257568	-3.1		
TGME49_309330	-4.6	TGME49_283882	-3.8	TGME49_280465	-3.1		
TGME49_243100	-4.6	TGME49_294330	-3.8	TGME49_283490	-3.1		
TGME49_254890	-4.6	TGME49_237800	-3.7	TGME49_316420	-3.1		
TGME49_315380	-4.6	TGME49_204500	-3.7	TGME49_297680	-3.1		
TGME49_297090	-4.6	TGME49_301160	-3.7	TGME49_217390	-3.0		
TGME49_301150	-4.6	TGME49_300990	-3.7	TGME49_266500	-3.0		
TGME49_209985	-4.6	TGME49_306235	-3.7	TGME49_232580	-3.0		
TGME49_318880	-4.6	TGME49_229240	-3.7	TGME49_262470	-3.0		

Additional file 2: Table S1B

Significantly higher expressed genes in tachyzoites (see also Figure 2).

Descending order: gene IDs and log2-fold changes (DESeq values) are indicated.

Gene ID	fold-chng (log2)	Gene ID	fold-chng (log2)	Gene ID	fold-chng (log2)	Gene ID	fold-chng (log2)	Gene ID	fold-chng (log2)
TGME49_215980	8.7	TGME49_275460	6.1	TGME49_269420	5.0	TGME49_266970	4.2	TGME49_313852	3.4
TGME49_270250	8.6	TGME49_300642	6.1	TGME49_237900	5.0	TGME49_216710	4.1	TGME49_265290	3.4
TGME49_227620	8.6	TGME49_310340	6.1	TGME49_310390	5.0	TGME49_265100	4.1	TGME49_210100	3.4
TGME49_286450	8.4	TGME49_300645	6.1	TGME49_270700	5.0	TGME49_232030	4.1	TGME49_314310	3.4
TGME49_280570	8.2	TGME49_283540	6.1	TGME49_308096	4.9	TGME49_255710	4.1	TGME49_212275	3.3
TGME49_233460	8.2	TGME49_252090	6.1	TGME49_236670	4.9	TGME49_266910	4.1	TGME49_239640	3.3
TGME49_275440	8.2	TGME49_226260	6.1	TGME49_267740	4.9	TGME49_215970	4.1	TGME49_233920	3.3
TGME49_323320	8.1	TGME49_268860	6.1	TGME49_213635	4.9	TGME49_241240	4.1	TGME49_282055	3.3
TGME49_201780	8.1	TGME49_245770	6.0	TGME49_239600	4.9	TGME49_258618	4.1	TGME49_305050	3.3
TGME49_233480	8.0	TGME49_254070	6.0	TGME49_247400	4.9	TGME49_271580	4.1	TGME49_320190	3.3
TGME49_270240	8.0	TGME49_215530	6.0	TGME49_323600	4.9	TGME49_228620	4.1	TGME49_258220	3.3
TGME49_310780	8.0	TGME49_320540	6.0	TGME49_323700	4.9	TGME49_245432	4.1	TGME49_244930	3.3
TGME49_230160	8.0	TGME49_233450	6.0	TGME49_323800	4.9	TGME49_234180	4.0	TGME49_241310	3.3
TGME49_250710	7.8	TGME49_217951	6.0	TGME49_266890	4.9	TGME49_274030	4.0	TGME49_259670	3.3
TGME49_227280	7.8	TGME49_202030	6.0	TGME49_296231	4.9	TGME49_233380	4.0	TGME49_269417	3.3
TGME49_271050	7.8	TGME49_280580	6.0	TGME49_239740	4.9	TGME49_213610	4.0	TGME49_281460	3.3
TGME49_218520	7.8	TGME49_204130	5.9	TGME49_221840	4.9	TGME49_287430	4.0	TGME49_288000	3.3
TGME49_253180	7.7	TGME49_311510	5.9	TGME49_322020	4.8	TGME49_317820	4.0	TGME49_289218	3.3
TGME49_207210	7.7	TGME49_258462	5.9	TGME49_220640	4.8	TGME49_242260	4.0	TGME49_226540	3.3
TGME49_247520	7.7	TGME49_204530	5.9	TGME49_239755	4.8	TGME49_241300	4.0	TGME49_300055	3.3
TGME49_266080	7.7	TGME49_208730	5.9	TGME49_292275	4.8	TGME49_225150	4.0	TGME49_292110	3.3
TGME49_277080	7.7	TGME49_218740	5.9	TGME49_293470	4.7	TGME49_239580	4.0	TGME49_286670	3.3
TGME49_291890	7.5	TGME49_318660	5.9	TGME49_242250	4.7	TGME49_243370	3.9	TGME49_244690	3.2
TGME49_208030	7.5	TGME49_309930	5.9	TGME49_309590	4.7	TGME49_237230	3.9	TGME49_271015	3.2
TGME49_262050	7.5	TGME49_297910	5.9	TGME49_225830	4.7	TGME49_203740	3.9	TGME49_236890	3.2
TGME49_215220	7.5	TGME49_250955	5.9	TGME49_259020	4.7	TGME49_235580	3.9	TGME49_225280	3.2
TGME49_209810	7.5	TGME49_202830	5.8	TGME49_242310	4.7	TGME49_311840	3.9	TGME49_246485	3.2
TGME49_204050	7.5	TGME49_226860	5.8	TGME49_322130	4.7	TGME49_316510	3.9	TGME49_300220	3.2
TGME49_214940	7.4	TGME49_238160	5.8	TGME49_233870	4.7	TGME49_315962	3.9	TGME49_286928	3.2
TGME49_240060	7.3	TGME49_262970	5.8	TGME49_274140	4.6	TGME49_261780	3.9	TGME49_210682	3.2
TGME49_213050	7.3	TGME49_221590	5.8	TGME49_287460	4.6	TGME49_220630	3.9	TGME49_224935	3.2
TGME49_227810	7.3	TGME49_316710	5.8	TGME49_296340	4.6	TGME49_213820	3.9	TGME49_289222	3.2
TGME49_256792	7.2	TGME49_252080	5.8	TGME49_249230	4.6	TGME49_284310	3.9	TGME49_231960	3.2
TGME49_297280	7.2	TGME49_290970	5.8	TGME49_292280	4.6	TGME49_271610	3.8	TGME49_236220	3.2
TGME49_254720	7.2	TGME49_258458	5.8	TGME49_287235	4.6	TGME49_218750	3.8	TGME49_300647	3.2
TGME49_230705	7.2	TGME49_266900	5.8	TGME49_225290	4.6	TGME49_312540	3.8	TGME49_224460	3.2
TGME49_295960	7.1	TGME49_239770	5.7	TGME49_308840	4.6	TGME49_306490	3.8	TGME49_216720	3.2
TGME49_208740	7.1	TGME49_202020	5.7	TGME49_233530	4.6	TGME49_268765	3.8	TGME49_285870	3.2
TGME49_313910	7.1	TGME49_234165	5.7	TGME49_287470	4.6	TGME49_248900	3.8	TGME49_249425	3.2
TGME49_289920	7.0	TGME49_242090	5.7	TGME49_320780	4.6	TGME49_287440	3.8	TGME49_258200	3.2
TGME49_253330	7.0	TGME49_306860	5.7	TGME49_248820	4.6	TGME49_265255	3.8	TGME49_292350	3.2
TGME49_309760	7.0	TGME49_279350	5.7	TGME49_213067	4.5	TGME49_290870	3.8	TGME49_222940	3.2
TGME49_255260	7.0	TGME49_282020	5.7	TGME49_229680	4.5	TGME49_289850	3.8	TGME49_293430	3.1
TGME49_214575	6.9	TGME49_291960	5.7	TGME49_297340	4.5	TGME49_203880	3.8	TGME49_209770	3.1
TGME49_211920	6.9	TGME49_200375	5.7	TGME49_238165	4.5	TGME49_309540	3.8	TGME49_241130	3.1
TGME49_293780	6.9	TGME49_208450	5.6	TGME49_299780	4.5	TGME49_244950	3.8	TGME49_280460	3.1
TGME49_209755	6.9	TGME49_233030	5.6	TGME49_215360	4.5	TGME49_221400	3.8	TGME49_203560	3.1
TGME49_271570	6.9	TGME49_219730	5.6	TGME49_220650	4.5	TGME49_273860	3.8	TGME49_284420	3.1
TGME49_293790	6.8	TGME49_250950	5.6	TGME49_215540	4.5	TGME49_323500	3.8	TGME49_252330	3.1
TGME49_251180	6.8	TGME49_314430	5.6	TGME49_266150	4.5	TGME49_286040	3.8	TGME49_272370	3.1
TGME49_211290	6.8	TGME49_300052	5.6	TGME49_252640	4.5	TGME49_293020	3.7	TGME49_293030	3.1
TGME49_311100	6.8	TGME49_314695	5.5	TGME49_237894	4.5	TGME49_320150	3.7	TGME49_279100	3.1
TGME49_245490	6.7	TGME49_200385	5.5	TGME49_292960	4.5	TGME49_308970	3.7	TGME49_266770	3.1
TGME49_275470	6.7	TGME49_272380	5.5	TGME49_278840	4.5	TGME49_230180	3.7	TGME49_226110	3.1
TGME49_271590	6.7	TGME49_251170	5.5	TGME49_297647	4.5	TGME49_289270	3.7	TGME49_243330	3.0
TGME49_200360	6.7	TGME49_323000	5.5	TGME49_224630	4.5	TGME49_201690	3.7	TGME49_245560	3.0
TGME49_212030	6.7	TGME49_265120	5.5	TGME49_224470	4.5	TGME49_212900	3.7	TGME49_295472	3.0
TGME49_203290	6.7	TGME49_244670	5.5	TGME49_203875	4.5	TGME49_210950	3.6	TGME49_252065	3.0
TGME49_288650	6.6	TGME49_219670	5.5	TGME49_224180	4.5	TGME49_318240	3.6	TGME49_236990	3.0
TGME49_205250	6.6	TGME49_259205	5.5	TGME49_217490	4.5	TGME49_208370	3.6	TGME49_241710	3.0
TGME49_281590	6.6	TGME49_220240	5.5	TGME49_276860	4.5	TGME49_242760	3.6	TGME49_236980	3.0
TGME49_203790	6.6	TGME49_252390	5.4	TGME49_208570	4.4	TGME49_308090	3.6		
TGME49_322030	6.6	TGME49_244260	5.4	TGME49_266920	4.4	TGME49_205430	3.6		
TGME49_247530	6.6	TGME49_244280	5.4	TGME49_318650	4.4	TGME49_323110	3.6		
TGME49_208830	6.6	TGME49_225120	5.4	TGME49_219110	4.4	TGME49_232110	3.6		
TGME49_200010	6.6	TGME49_227380	5.4	TGME49_309530	4.4	TGME49_292375	3.6		
TGME49_275860	6.6	TGME49_229220	5.4	TGME49_226880	4.4	TGME49_299220	3.6		
TGME49_247440	6.6	TGME49_211460	5.3	TGME49_218955	4.4	TGME49_297492	3.6		
TGME49_258870	6.5	TGME49_292270	5.3	TGME49_201260	4.4	TGME49_252500	3.6		
TGME49_212270	6.5	TGME49_235140	5.3	TGME49_237015	4.4	TGME49_218460	3.5		
TGME49_204340	6.5	TGME49_245428	5.3	TGME49_229010	4.4	TGME49_257360	3.5		
TGME49_262730	6.5	TGME49_323010	5.3	TGME49_220890	4.4	TGME49_295750	3.5		
TGME49_293620	6.4	TGME49_286770	5.3	TGME49_252350	4.3	TGME49_236010	3.5		
TGME49_223430	6.4	TGME49_202025	5.3	TGME49_215885	4.3	TGME49_272265	3.5		
TGME49_323020	6.4	TGME49_237893	5.3	TGME49_317830	4.3	TGME49_310260	3.5		
TGME49_225560	6.4	TGME49_210600	5.3	TGME49_298590	4.3	TGME49_265130	3.5		
TGME49_258622	6.3	TGME49_315802	5.2	TGME49_316560	4.3	TGME49_273510	3.5		
TGME49_296020	6.3	TGME49_245530	5.2	TGME49_227630	4.3	TGME49_272285	3.5		
TGME49_225160	6.3	TGME49_304920	5.2	TGME49_218450	4.3	TGME49_268790	3.5		
TGME49_226380	6.3	TGME49_261740	5.2	TGME49_210370	4.3	TGME49_230470	3.5		
TGME49_316780	6.3	TGME49_268360	5.2	TGME49_250115	4.3	TGME49_215520	3.5		
TGME49_264660	6.3	TGME49_260400	5.1	TGME49_216140	4.3	TGME49_275320	3.5		
TGME49_322110	6.3	TGME49_308093	5.1	TGME49_207650	4.3	TGME49_264140	3.5		
TGME49_323310	6.3	TGME49_239780	5.1	TGME49_254790	4.3	TGME49_247220	3.5		
TGME49_301400	6.3	TGME49_258540	5.1	TGME49_287240	4.3	TGME49_276200	3.5		
TGME49_295950	6.3	TGME49_287450	5.1	TGME49_248730	4.2	TGME49_203000	3.5		
TGME49_247030	6.3	TGME49_292390	5.1	TGME49_254470	4.2	TGME49_213660	3.5		
TGME49_216160	6.3	TGME49_278450	5.1	TGME49_250670	4.2	TGME49_235590	3.5		
TGME49_236870	6.3	TGME49_294200	5.1	TGME49_278680	4.2	TGME49_227430	3.4		
TGME49_290700	6.2	TGME49_216770	5.1	TGME49_240470	4.2	TGME49_218960	3.4		
TGME49_202620	6.2	TGME49_228065	5.1	TGME49_263750	4.2	TGME49_288440	3.4		
TGME49_253690	6.2	TGME49_314080	5.1	TGME49_230820	4.2	TGME49_307760	3.4		
TGME49_285250	6.2	TGME49_210678	5.0	TGME49_259900	4.2	TGME49_247195	3.4		
TGME49_295935	6.2	TGME49_323200	5.0	TGME49_243360	4.2	TGME49_225790	3.4		
TGME49_245980	6.2	TGME49_313050	5.0	TGME49_250500	4.2	TGME49_266100	3.4		
TGME49_258580	6.1	TGME49_291040	5.0	TGME49_249300	4.2	TGME49_204395	3.4		
TGME49_305250	6.1	TGME49_211630	5.0	TGME49_220950	4.2	TGME49_307470	3.4		
TGME49_300048	6.1	TGME49_296121	5.0	TGME49_278080	4.2	TGME49_254160	3.4		

Additional file 2: Table S2

List of annotated Family A genes. The color key for the expression heat map (log2 fold change) is included.

Description	ID	Merozoites (RPKM)	Tachyzoites (RPKM)	log2 fold change (DESeq)	padj	Heatmap
Toxoplasma gondii family A protein	TGME49_243150	421	11	-6	4.82E-20	
Toxoplasma gondii family A protein	TGME49_278360	195	5	-5	1.82E-30	
Toxoplasma gondii family A protein	TGME49_278320	352	11	-5	1.44E-29	
Toxoplasma gondii family A protein	TGME49_243140	140	5	-5	3.49E-27	
Toxoplasma gondii family A protein	TGME49_243180	281	9	-5	1.61E-28	
Toxoplasma gondii family A protein	TGME49_278340	412	14	-5	3.24E-21	
Toxoplasma gondii family A protein	TGME49_243130	139	5	-5	4.29E-27	
Toxoplasma gondii family A protein	TGME49_278420	115	4	-5	1.29E-14	
Toxoplasma gondii family A protein	TGME49_243160	150	6	-5	8.44E-26	
Toxoplasma gondii family A protein	TGME49_278380	156	7	-5	1.14E-15	
Toxoplasma gondii family A protein	TGME49_243120	115	5	-5	7.67E-24	
Toxoplasma gondii family A protein	TGME49_278390	150	7	-5	1.14E-20	
Toxoplasma gondii family A protein	TGME49_278290	94	4	-5	6.55E-07	
Toxoplasma gondii family A protein	TGME49_278350	69	3	-5	3.33E-13	
Toxoplasma gondii family A protein	TGME49_278370	167	8	-5	2.35E-07	
Toxoplasma gondii family A protein	TGME49_278430	164	8	-5	4.83E-06	
Toxoplasma gondii family A protein	TGME49_243100	167	8	-5	5.18E-23	
Toxoplasma gondii family A protein	TGME49_278410	151	8	-5	2.70E-09	
Toxoplasma gondii family A protein	TGME49_243170	99	5	-5	4.64E-22	
Toxoplasma gondii family A protein	TGME49_278330	51	3	-5	3.90E-10	
Toxoplasma gondii family A protein	TGME49_266340	263	15	-4	1.59E-19	
Toxoplasma gondii family A protein	TGME49_266335	233	14	-4	3.01E-14	
Toxoplasma gondii family A protein	TGME49_278090	172	13	-4	1.99E-04	
Toxoplasma gondii family A protein	TGME49_243190	339	28	-4	1.88E-18	
Toxoplasma gondii family A protein	TGME49_278300	21	2	-4	4.37E-06	
Toxoplasma gondii family A protein	TGME49_278400	75	8	-4	3.00E-04	
Toxoplasma gondii family A protein	TGME49_278100	204	22	-4	5.80E-06	
Toxoplasma gondii family A protein	TGME49_266330	84	9	-4	9.67E-14	
Toxoplasma gondii family A protein	TGME49_327300	241	27	-3	7.01E-03	
Toxoplasma gondii family A protein	TGME49_278070	86	26	-2	1.71E-01	
Toxoplasma gondii family A protein	TGME49_242900	38	12	-2	1.84E-05	
Toxoplasma gondii family A protein	TGME49_278365	104	36	-2	5.66E-03	
Toxoplasma gondii family A protein	TGME49_278080	3	64	4	8.97E-09	

		log2 fold change
Mero		≤ -7
		≤ -6
		≤ -5
		≤ -4
		≤ -3
		3 > x > -3
		≥ 3
		≥ 4
		≥ 5
		≥ 6
Tachy		≥ 7

Additional file 2: Table S3

Differential expression of KRUF family genes

ID	Description	Merozoites (RPKM)	Tachyzoites (RPKM)	log2 fold chng (DESeq)	padj	Heatmap
TGME49_251180	KRUF family protein	1	93	6.8	2.66E-34	
TGME49_323020	KRUF family protein	1	81	6.4	1.69E-34	
TGME49_295950	KRUF family protein	1	77	6.3	1.85E-33	
TGME49_295935	KRUF family protein	1	112	6.2	3.29E-35	
TGME49_250955	KRUF family protein	2	132	5.9	3.11E-32	
TGME49_250950	KRUF family protein	2	96	5.6	1.05E-29	
TGME49_251170	KRUF family protein	2	115	5.5	5.74E-29	
TGME49_323000	KRUF family protein	2	107	5.5	3.60E-29	
TGME49_210600	KRUF family protein	4	170	5.3	5.37E-28	
TGME49_292390	KRUF family protein	4	168	5.1	1.38E-26	
TGME49_292375	KRUF family protein	5	68	3.6	9.69E-15	
TGME49_252065	KRUF family protein	11	107	3.0	4.10E-11	
TGME49_252190	KRUF family protein	12	90	2.6	1.29E-08	
TGME49_252070	KRUF family protein	23	138	2.3	5.30E-07	

		log2 fold change
Mero		≤-7
		≤-6
		≤-5
		≤-4
		≤ -3
		3>x>-3
		≥3
		≥4
		≥5
		≥6
Tachy		≥7

Additional file 2: Table S4

List of definitively and provisionally annotated rhoptry genes. The color key for the expression heat map (log2 fold change) is included.

ID	Description	Merozoites (RPKM)	Tachyzoites (RPKM)	log2 fold change (DESeq)	padj	Heat map
TGME49_262050	ROP39	1	270	7.5	5.23E-47	
TGME49_227810	ROP11	1	154	7.3	7.23E-44	
TGME49_253330	BPK1	2	278	7.0	1.74E-16	
TGME49_211290	ROP15	3	445	6.8	1.77E-41	
TGME49_205250	ROP18	3	401	6.6	1.53E-39	
TGME49_262730	ROP16	1	162	6.5	1.13E-37	
TGME49_296020	ROP2L11	0	46	6.3	5.47E-27	
TGME49_258580	ROP17	4	361	6.1	3.72E-35	
TGME49_291960	ROP40	6	373	5.7	3.67E-31	
TGME49_261740	ROP47	39	1805	5.2	1.61E-28	
TGME49_308093	Incomplete ROPK	2	103	5.1	1.03E-24	
TGME49_308096	Incomplete ROPK	2	92	4.9	3.96E-23	
TGME49_239600	ROP23	1	32	4.9	5.34E-22	
TGME49_242250	ROP19B	5	180	4.7	1.01E-19	
TGME49_309590	ROP1	39	1299	4.7	5.39E-25	
TGME49_308090	ROP5	78	1160	3.6	1.65E-15	
TGME49_252500	ROP47(Polo kinase)	4	55	3.6	8.56E-14	
TGME49_230470	ROP46	1	19	3.5	6.56E-07	
TGME49_266100	ROP41	2	32	3.4	3.86E-12	
TGME49_258370	ROP28	0	3	2.9	5.34E-04	
TGME49_295105	Putative ROP	166	1442	2.8	1.75E-10	
TGME49_252360	ROP24	23	201	2.8	7.73E-10	
TGME49_242230	ROP29	40	301	2.6	9.75E-09	
TGME49_242118	degraded ROPK	40	303	2.6	1.04E-08	
TGME49_234950	Incomplete ROPK	4	28	2.5	5.36E-07	
TGME49_243730	ROP9	48	338	2.5	1.46E-08	
TGME49_312270	ROP13	19	79	1.8	1.36E-04	
TGME49_315210	Putative ROP	32	134	1.8	1.16E-04	
TGME49_215785	ROP2A	749	2924	1.7	3.69E-04	
TGME49_304740	ROP35	26	80	1.3	5.45E-03	
TGME49_215775	ROP8	654	2004	1.3	6.50E-03	
TGME49_295110	ROP7	714	2120	1.3	7.93E-03	
TGME49_211260	ROP26	56	160	1.2	1.42E-02	
TGME49_315490	ROP10	50	128	1.1	3.86E-02	

TGME49_295125	ROP4	969	2422	1.0	3.82E-02	
TGME49_242110	ROP38	169	386	0.9	8.16E-02	
TGME49_242240	ROP19A	96	216	0.9	1.10E-01	
TGME49_315940	Putative ROP	9	20	0.9	1.20E-01	
TGME49_201140	ROP33A	23	50	0.8	9.67E-02	
TGME49_315220	ROP14	38	72	0.6	2.99E-01	
TGME49_218270	ROP48	56	104	0.6	3.13E-01	
TGME49_258660	ROP6	218	393	0.6	3.45E-01	
TGME49_258800	ROP31	15	27	0.5	4.64E-01	
TGME49_240090	ROP34	50	81	0.4	5.02E-01	
TGME49_294560	ROP37	34	43	0.0	1.00E+00	
TGME49_258230	ROP20	30	37	0.0	9.80E-01	
TGME49_203990	ROP12	231	225	-0.4	7.95E-01	
TGME49_249470	truncated ROPK	22	20	-0.4	5.16E-01	
TGME49_201130	ROP33	47	39	-0.6	3.19E-01	
TGME49_313330	ROP27	25	20	-0.6	2.63E-01	
TGME49_202780	ROP25	33	25	-0.7	2.16E-01	
TGME49_227010	ROP30	15	11	-0.7	2.74E-01	
TGME49_207700	ROP22	8	5	-0.8	2.26E-01	
TGME49_296000	ROP2L12	22	15	-0.9	1.12E-01	
TGME49_270920	ROP32	60	27	-1.5	2.24E-03	
TGME49_281675	ROP45	70	31	-1.5	2.69E-02	
TGME49_263220	ROP21	106	36	-1.9	3.01E-05	
TGME49_207610	ROP36	18	4	-2.4	4.22E-06	
TGME49_321700	ROPK fragment	752	68	-3.8	1.97E-09	
TGME49_321710	ROP44	697	61	-3.8	1.06E-11	
TGME49_210095	ROP43	549	42	-4.0	3.17E-18	
TGME49_209985	ROP42	1241	63	-4.6	1.21E-24	
TGME49_274170	Incomplete ROPK	106.49	4.38	-4.9	2.68E-14	

		log2 fold change
**Mero**		**≤ -7**
		**≤ -6**
		**≤ -5**
		**≤ -4**
		**≤ -3**
		**3 > x > -3**
		**≥ 3**
		**≥ 4**
		**≥ 5**
		**≥ 6**
**Tachy**		**≥ 7**

Additional file 2: Table S5

Protease inhibitors including differentially expressed secreted KAZAL-type proteins. The color key for the expression heat map (log2 fold change) is included.

ID	Description	Merozoites (RPKM)	Tachyzoites (RPKM)	log2 fold change (DESeq)	padj	Heatmap
TGME49_266600	Kazal-type serine protease inhibitor domain-containing protein	941	26	-5.5	2.17E-30	
TGME49_283470	Kazal-type serine protease inhibitor domain-containing protein	120	7	-4.5	3.13E-05	
TGME49_259890	Kazal-type serine protease inhibitor domain-containing protein	168	20	-3.4	1.72E-14	
TGME49_266610	Kazal-type serine protease inhibitor domain-containing protein	60	11	-2.7	6.76E-10	
TGME49_224080	Kazal-type serine protease inhibitor domain-containing protein	3	19	2.1	1.08E-04	
TGME49_217430	protease inhibitor PI1 (PI1)	48	24	-1.3	2.58E-02	
TGME49_208450	protease inhibitor PI2 (PI2)	3	168	5.6	5.38E-30	
TGME49_208430	serine proteinase inhibitor PI-2, putative	88	26	-2.1	3.16E-06	
TGME49_246130	serpin (serine proteinase inhibitor) superfamily protein	548	110	-2.6	4.67E-06	

		log2 fold change
Mero		≤ -7
		≤ -6
		≤ -5
		≤ -4
		≤ -3
		3 > x > -3
		≥ 3
		≥ 4
		≥ 5
		≥ 6
Tachy		≥ 7
