# Supplementary material for: Asexual expansion of Toxoplasma gondii merozoites is distinct from tachyzoites and entails expression of non-overlapping gene families to attach, invade, and replicate within feline enterocytes
Source: BMC Genomics. 2015 Feb 13;16(1):66. doi: 10.1186/s12864-015-1225-x (PMC4340605; doi:10.1186/s12864-015-1225-x)

Additional file 2: Figure S1

Differential expression of – *T. gondii* Family A-E genes. (A) Bar graph of Family A gene expression. (B) Heat map showing levels of expression for members of all five families (RPKM).(C) Bar graph of Family D gene expression. Differentially expressed genes (>log2 [3]) are indicated with asterisks.

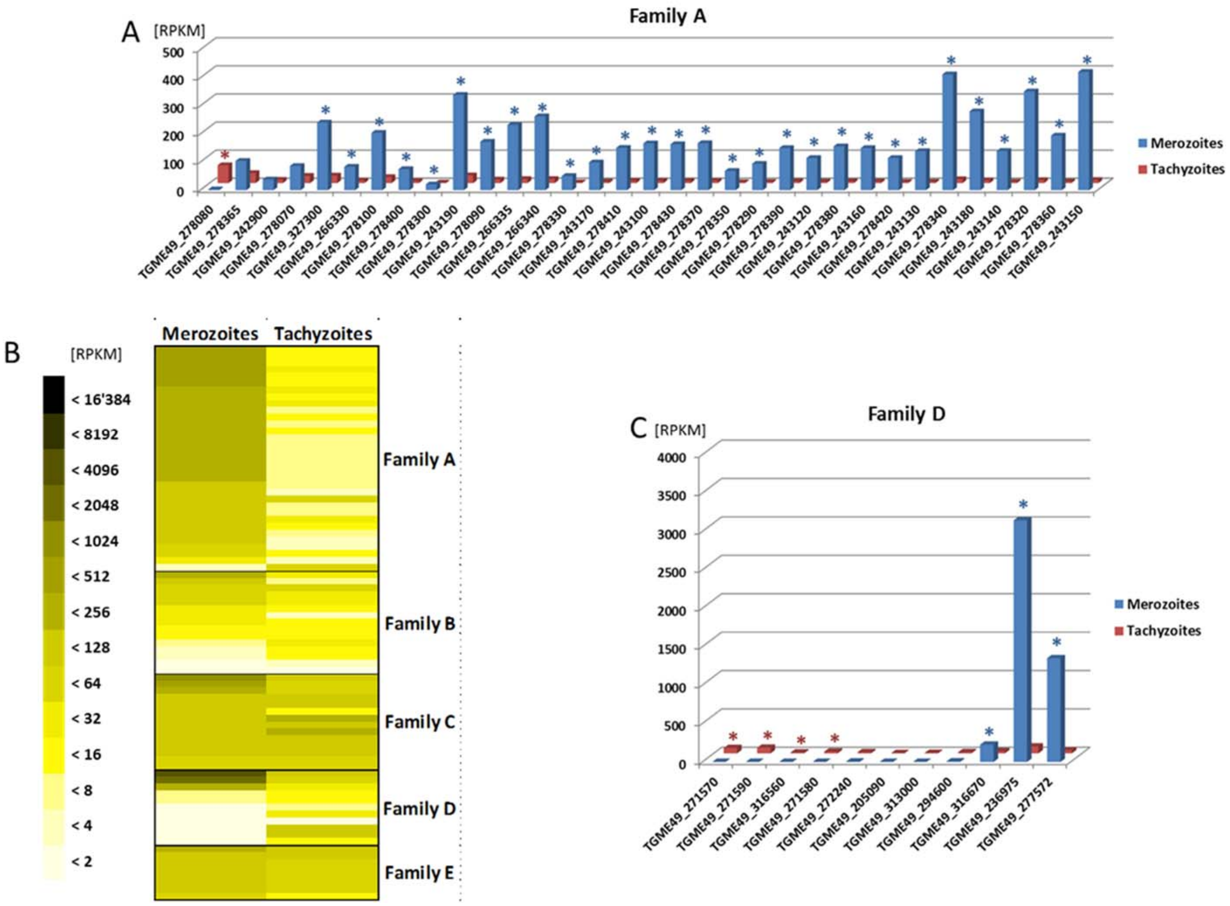

Additional file 2: Figure S2

Differential expression of metabolic genes. Significantly regulated genes contained in the iCS382 list used for constructing the metabolic model for *Toxoplasma* . (A) Bar graph showing RPKM values only for the 18 metabolic genes whose differences in expression was >log2[3] . (B) Corresponding detailed list of regulated metabolic genes including adjusted p-values. Higher expression in tachyzoites (pink background), in merozoites (blue background).

A

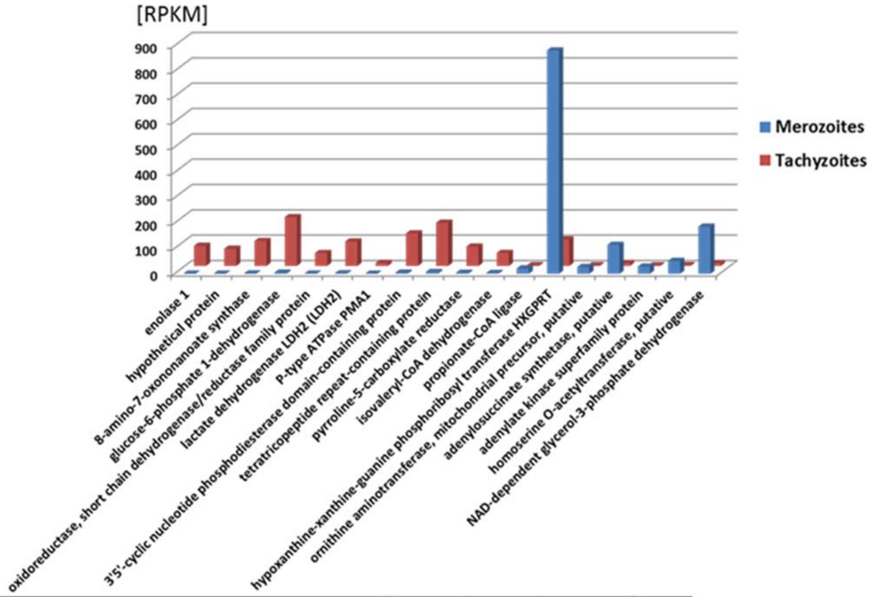

B

ID	Description	Merozoites RPKM	Tachyzoites RPKM	log2 fold change (DESeq)	padj
TGME49_268860	enolase 1	1	80	6.1	1.74E-07
TGME49_202030	hypothetical protein	1	68	6.0	1.99E-33
TGME49_290970	8-amino-7-oxononanoate synthase	1	99	5.8	1.23E-06
TGME49_294200	glucose-6-phosphate 1-dehydrogenase	5	193	5.1	9.67E-27
TGME49_313050	oxidoreductase, short chain dehydrogenase/reductase family protein	1	51	5.0	2.34E-22
TGME49_291040	lactate dehydrogenase LDH2 (LDH2)	2	97	5.0	1.64E-09
TGME49_252640	P-type ATPase PMA1	0	12	4.5	9.08E-12
TGME49_266920	3'5'-cyclic nucleotide phosphodiesterase domain-containing protein	5	129	4.4	6.89E-22
TGME49_216140	tetratricopeptide repeat-containing protein	7	171	4.3	3.95E-17
TGME49_271610	pyrroline-5-carboxylate reductase	4	77	3.8	4.81E-15
TGME49_205430	isovaleryl-CoA dehydrogenase	3	52	3.6	7.89E-13
TGME49_232580	propionate-CoA ligase	21	3	-3.0	2.64E-10
TGME49_200320	hypoxanthine-xanthine-guanine phosphoribosyl transferase HXGPRT	882	105	-3.4	1.12E-09
TGME49_269110	ornithine aminotransferase, mitochondrial precursor, putative	27	3	-3.5	4.59E-12
TGME49_279450	adenylosuccinate synthetase, putative	115	12	-3.6	6.51E-12
TGME49_281970	adenylate kinase superfamily protein	29	2	-3.8	2.42E-01
TGME49_320730	homoserine O-acetyltransferase, putative	51	4	-4.0	2.17E-16
TGME49_210260	NAD-dependent glycerol-3-phosphate dehydrogenase	186	12	-4.3	2.70E-21

Additional file 2: Figure S3

Differential expression of *SRS* genes. (A) Heat maps with color-coded RPKM values. Left side: ordered for descending RPKMs in merozoites. Right side: ordered for descending RPKMs in tachyzoites. (B) Scatter plot of RPKM values for all 111 annotated *SRS*s.

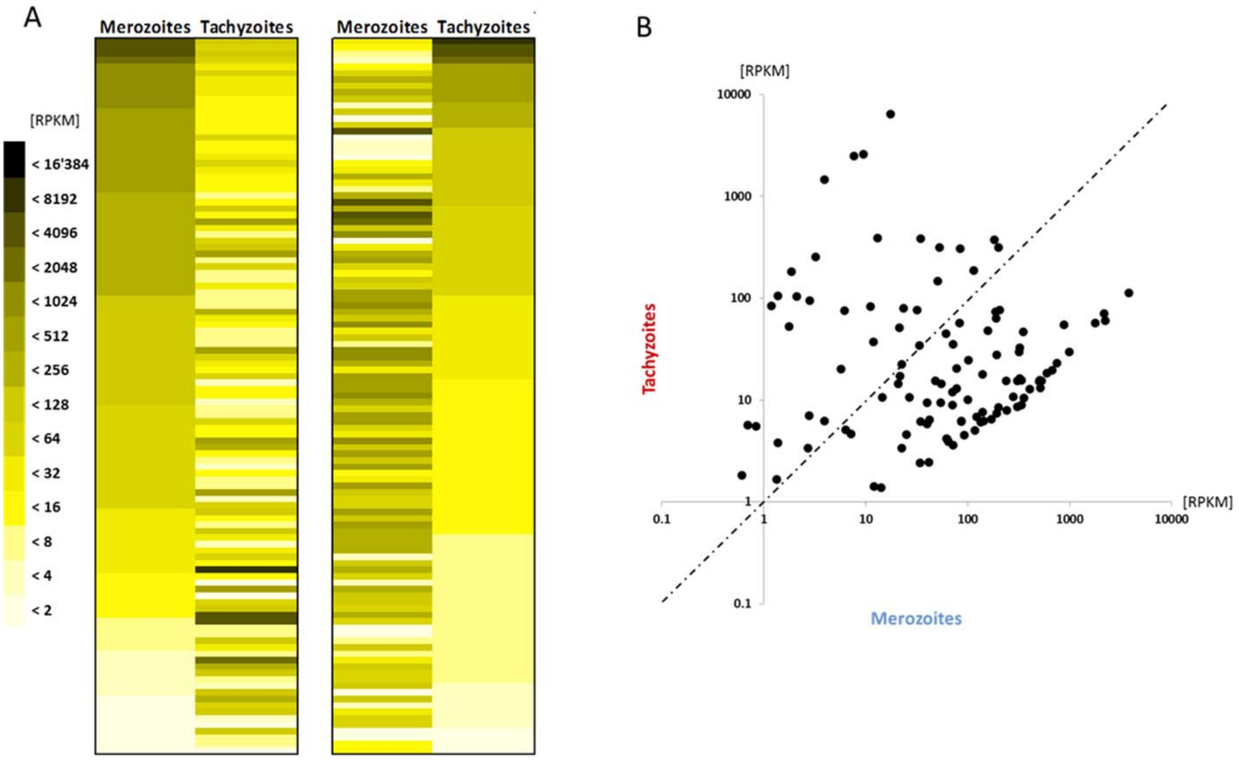

Additional file 2: Figure S4

Differential expression of microneme (*MIC*) genes annotated in ToxoDB. (A) Bar graph showing RPKM values for all *MIC*s (differentially expressed genes (>log2 [3]) are indicated with asterisks). (B) Corresponding detailed list of *MIC* genes including adjusted p-values. Higher expression in tachyzoites (pink background), in merozoites (blue background).

B

ID	Description	Merozoites RPKM	Tachyzoites RPKM	log2 fold change (DESeq)	padj
TGME49_201780	MIC2	2	510	8.1	2.22E-52
TGME49_250710	MIC10	2	607	7.8	1.18E-50
TGME49_218520	MIC6	2	512	7.8	1.09E-49
TGME49_277080	MIC5	4	975	7.7	2.89E-49
TGME49_291890	MIC1	5	1189	7.5	1.67E-48
TGME49_208030	MIC4	3	774	7.5	2.74E-47
TGME49_214940	MIC2AP	3	569	7.4	2.74E-47
TGME49_208740	Putative MIC	1	171	7.1	9.98E-17
TGME49_245490	MIC8	1	166	6.7	3.94E-40
TGME49_204530	MIC11	14	984	5.9	1.79E-32
TGME49_208730	Putative MIC	2	164	5.9	2.36E-07
TGME49_261780	MIC7	9	170	3.9	6.34E-17
TGME49_247195	MIC15	3	41	3.4	3.86E-14
TGME49_289630	MIC16	5	46	3.0	2.31E-10
TGME49_319560	MIC3	205	1629	2.7	1.30E-09
TGME49_218310	MIC14	48	58	0.0	9.60E-01
TGME49_260190	MIC13	41	40	-0.3	5.93E-01
TGME49_286740	MIC-like	45	27	-1.0	3.91E-02
TGME49_275798	Putative MIC	68	17	-2.3	4.46E-07
TGME49_245485	MIC9	283	45	-2.9	2.04E-11
TGME49_244180	MIC-like	86	9	-3.6	2.60E-15
TGME49_200230	MIC17C	4263	341	-3.9	2.38E-19
TGME49_200240	MIC17B	7428	549	-4.1	2.83E-20
TGME49_200250	MIC17A	9188	532	-4.4	3.67E-23
TGME49_254430	Putative MIC	647	30	-4.7	1.10E-11
TGME49_267680	MIC12	1509	56	-5.0	2.05E-28

A

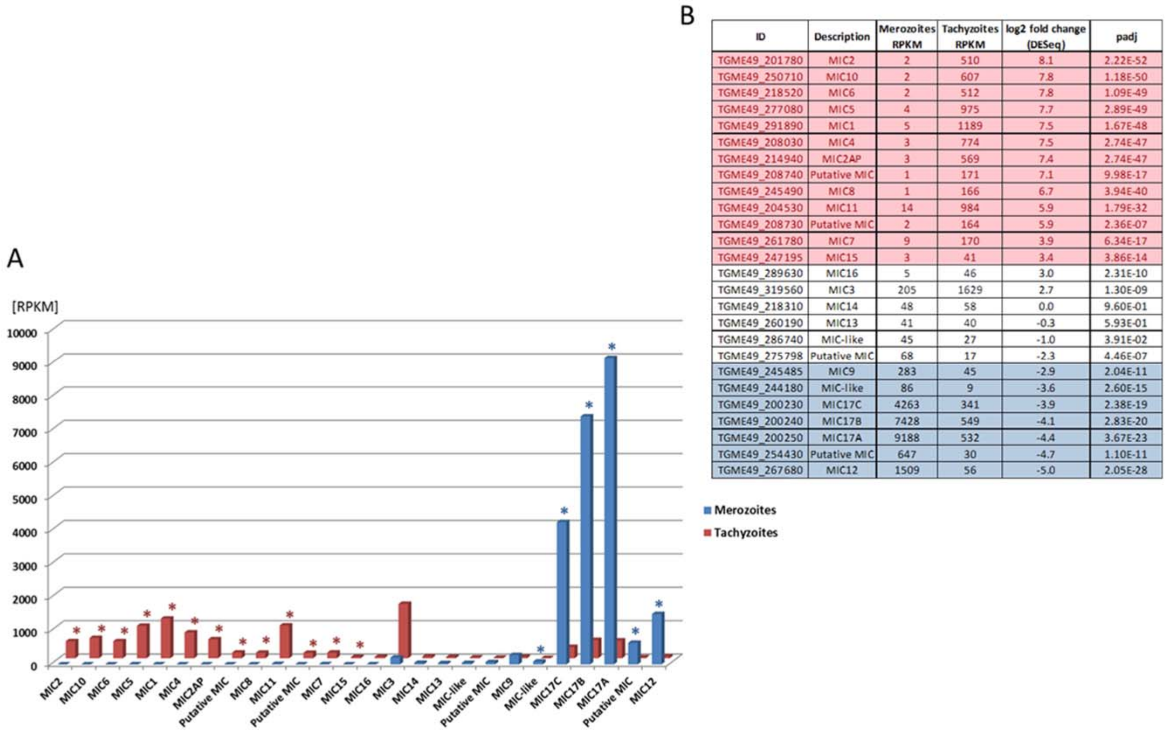

Additional file 2: Figure S5

Differential expression of genes coding for PAN-domain containing proteins. (A) List representation of 13 genes ordered by increasing stage-specificity of expression. Genes with a significantly higher expression (>log2 [3]) in merozoites are highlighted with blue. (B) Bar graph of RPKM values for all PAN-domain genes. Asterisk: >log2 [3] difference in expression in merozoites.

A

Description	ID	Merozoites RPKM	Tachyzoites RPKM	log2 fold change (DESeq)	padj
PAN domain-containing protein	TGME49_209920	7	8	0.0	1.00E+00
PAN domain-containing protein	TGME49_235183	48	60	0.0	9.44E-01
PAN domain-containing protein	TGME49_249150	68	36	-1.2	4.01E-01
PAN domain-containing protein	TGME49_235200	258	80	-2.0	1.78E-04
PAN domain-containing protein	TGME49_235315	239	66	-2.2	6.61E-05
PAN domain-containing protein	TGME49_209180	139	24	-2.8	5.15E-09
PAN/Apple domain-containing protein	TGME49_286150	150	16	-3.5	4.28E-09
microneme protein MIC17C	TGME49_200230	4263	341	-3.9	2.38E-19
microneme protein MIC17B	TGME49_200240	7428	549	-4.1	2.83E-20
PAN/Apple domain-containing protein	TGME49_200270	5730	418	-4.1	1.50E-14
microneme protein MIC17A	TGME49_200250	9188	532	-4.4	3.67E-23
PAN domain-containing protein	TGME49_235390	277	13	-4.7	2.57E-12
PAN domain-containing protein	TGME49_232400	2318	64	-5.5	5.35E-32

B

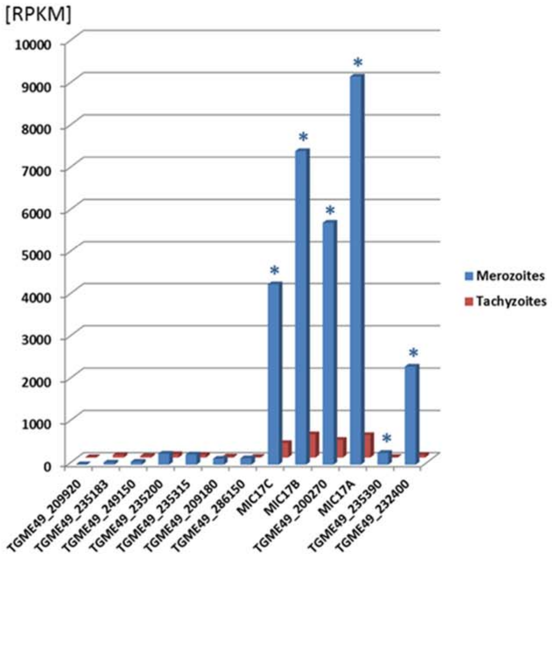

Additional file 2: Figure S6

Differential expression of annotated rhoptry (*ROP*) genes. (A) Scatter plot of RPKM values for all annotated genes (see also Additional file 2: Table S4). (B) Bar graph of RPKM values for all *ROP* genes. Asterisk: >log2 [3] difference in expression. Y-axis: RPKM values. (C) List of differentially expressed genes only. Higher expression in tachyzoites (pink background), in merozoites (blue background).

A

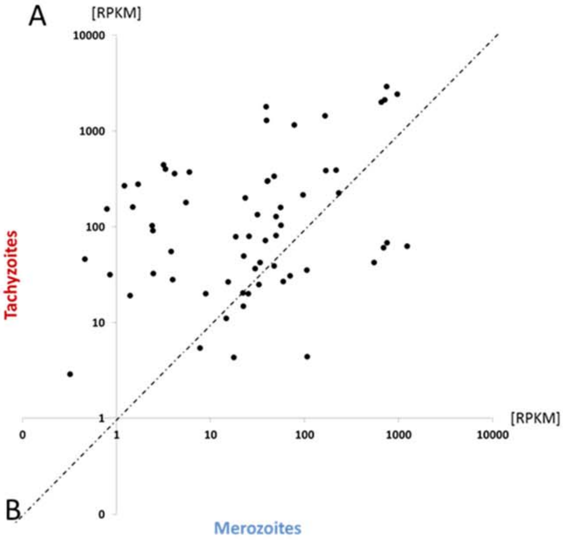

C

ID	Description	Merozoites (RPKM)	Tachyzoites (RPKM)	log2 fold change (DESeq)	padj
TGME49_262050	ROP39	1	270	7.5	5.23E-47
TGME49_227810	ROP11	1	154	7.3	7.23E-44
TGME49_253330	BPK1	2	278	7.0	1.74E-16
TGME49_211290	ROP15	3	445	6.8	1.77E-41
TGME49_205250	ROP18	3	401	6.6	1.53E-39
TGME49_262730	ROP16	1	162	6.5	1.13E-37
TGME49_296020	ROP2L11	0	46	6.3	5.47E-27
TGME49_258580	ROP17	4	361	6.1	3.72E-35
TGME49_291960	ROP40	6	373	5.7	3.67E-31
TGME49_261740	ROP47	39	1805	5.2	1.61E-28
TGME49_308093	Incomplete ROPK	2	103	5.1	1.03E-24
TGME49_308096	Incomplete ROPK	2	92	4.9	3.96E-23
TGME49_239600	ROP23	1	32	4.9	5.34E-22
TGME49_242250	ROP19B	5	180	4.7	1.01E-19
TGME49_309590	ROP1	39	1299	4.7	5.39E-25
TGME49_308090	ROP5	78	1160	3.6	1.65E-15
TGME49_252500	ROP47	4	55	3.6	8.56E-14
TGME49_230470	ROP46	1	19	3.5	6.56E-07
TGME49_266100	ROP41	2	32	3.4	3.86E-12
TGME49_321700	ROPK fragment	752	68	-3.8	1.97E-09
TGME49_321710	ROP44	697	61	-3.8	1.06E-11
TGME49_210095	ROP43	549	42	-4.0	3.17E-18
TGME49_209985	ROP42	1241	63	-4.6	1.21E-24
TGME49_274170	Incomplete ROPK	106	4	-4.9	2.68E-14

B

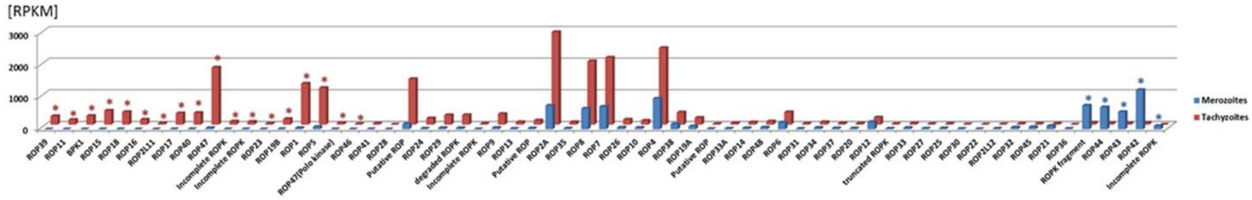

Additional file 2: Figure S7

Differential expression of annotated dense granule (*GRA*) genes. (A) Scatter plot of RPKM values for all genes.(B) Bar graph of RPKM values for all *GRA* genes. Asterisk: >log2 [3] difference in expression. (C) List of all annotated *GRA*s: higher expression in tachyzoites (pink background), in merozoites (blue background).

A

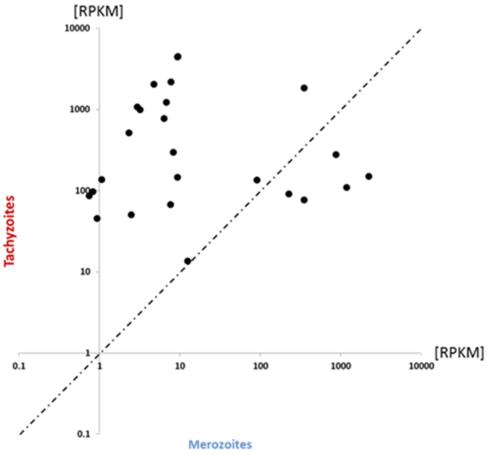

ID	Description	Merozoites (RPKM)	Tachyzoites (RPKM)	log2 fold change (DESeq)	padj
TGME49_270250	GRA1	9	4476	8.6	1.34E-57
TGME49_227620	GRA2	9	4514	8.6	1.62E-57
TGME49_286450	GRA5	5	2058	8.4	2.31E-56
TGME49_275440	GRA6	3	1081	8.2	1.58E-54
TGME49_310780	GRA4	3	993	8.0	3.02E-52
TGME49_227280	GRA3	8	2188	7.8	3.97E-51
TGME49_215220	GRA22	2	517	7.5	4.09E-48
TGME49_254720	GRA8	7	1226	7.2	2.04E-45
TGME49_275470	GRA15	1	137	6.7	2.88E-38
TGME49_288650	GRA12B	6	774	6.6	1.90E-40
TGME49_208830	GRA16	1	97	6.6	1.05E-37
TGME49_200010	GRA20	1	87	6.6	1.86E-37
TGME49_245428	GRA19A	1	45	5.3	3.67E-23
TGME49_245432	GRA19B	2	51	4.1	8.96E-17
TGME49_239740	GRA14	8	298	4.9	4.91E-25
TGME49_230180	GRA24	9	146	3.7	1.15E-15
TGME49_241610	GRA21	8	67	2.8	1.95E-09
TGME49_203310	GRA7	351	1837	2.1	3.08E-06
TGME49_251540	GRA9	90	136	0.3	6.42E-01
TGME49_268900	GRA10	12	14	-0.2	8.34E-01
TGME49_275850	GRA12A	227	91	-1.6	3.49E-04
TGME49_297880	GRA23	867	278	-1.9	1.22E-05
TGME49_222170	DG32	351	77	-2.5	1.51E-07
TGME49_237800	GRA11B	1187	110	-3.7	1.72E-17
TGME49_212410	GRA11A	2214	151	-4.2	2.02E-11

B

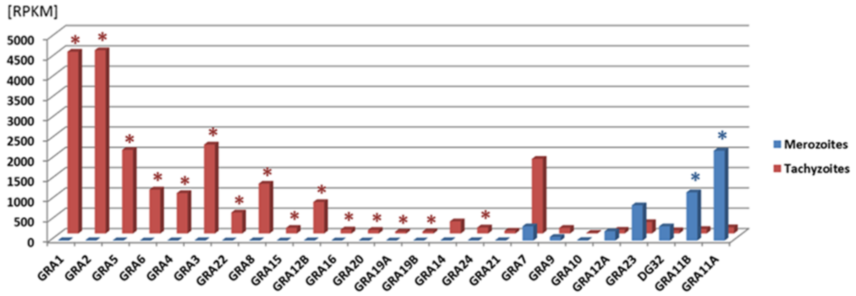

Additional file 2: Figure S8

Differential regulation of Tg*AP2* genes. **(**A) Stage-specific expression of Tg*AP2* genes. (B) Bar graph depicting percent expression of individual Tg*AP2* mRNAs in merozoites or tachyzoites ordered by –fold difference in expression starting with the *AP2XII1* and *AP2VIIA2* (TGME49_218960, TGME49_280460) with strongly tachyzoite-specific expression. *AP2VIIA1* was the only family member expressed >8-fold higher in merozoites. Asterisks: RPKM differences >8-fold.

A

ID	Description	Merozoites (RPKM)	Tachyzoites (RPKM)	log2 fold change (DESeq)	padj	Heatmap
TGME49_218960	AP2 domain transcription factor AP2XII-1 (AP2XII1)	3	45	3.4	3.30E-14	
TGME49_280460	AP2 domain transcription factor AP2VIIa-2 (AP2VIIA2)	7	75	3.1	1.50E-11	
TGME49_215340	AP2 domain transcription factor AP2X-10 (AP2X10)	13	102	2.7	4.13E-09	
TGME49_253380	AP2 domain transcription factor AP2III-2 (AP2III2)	3	28	2.7	4.92E-09	
TGME49_211720	AP2 domain transcription factor AP2IV-5 (AP2IV5)	6	46	2.6	1.42E-08	
TGME49_205650	AP2 domain transcription factor AP2VIIa-3 (AP2VIIA3)	16	83	2.1	4.09E-06	
TGME49_269010	AP2 domain transcription factor AP2VIII-7 (AP2VIII7)	14	71	2.0	7.01E-06	
TGME49_252370	AP2 domain transcription factor AP2III-1 (AP2III1)	3	17	2.0	3.56E-05	
TGME49_271030	AP2 domain transcription factor AP2VIII-6 (AP2VIII6)	8	35	1.9	4.92E-05	
54 other AP2 genes						
TGME49_318610	AP2 domain transcription factor AP2IV-3 (AP2IV3)	50	16	-1.9	2.10E-05	
TGME49_267460	AP2 domain transcription factor AP2IX-1 (AP2IX1)	38	9	-2.4	6.50E-08	
TGME49_224230	AP2 domain transcription factor AP2X-3 (AP2X3)	21	4	-2.9	1.24E-10	
TGME49_280470	AP2 domain transcription factor AP2VIIa-1 (AP2VIIA1)	69	11	-3.0	8.91E-12	

		log2 fold change
Mero		≤-7
		≤-6
		≤-5
		≤-4
		≤ -3
		3>x>-3
		≥3
		≥4
		≥5
		≥6
Tachy		≥7

B

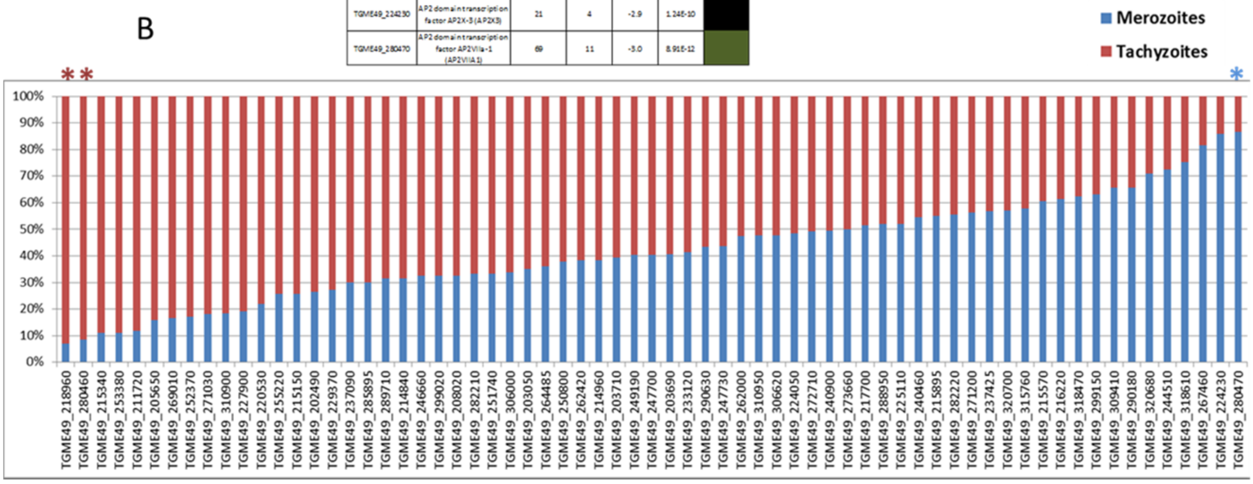

Supplement: Additional file 2: Figure S1- S8. — Differential expression of – T. gondii genes and gene families: S1. Differential expression of – T. gondii Family A-E genes. S2. Differential expression of metabolic genes. S3. Differential expression of SRS genes. S4. Differential expression of microneme (MIC) genes annotated in ToxoDB. S5. Differential expression of genes coding for PAN-domain containing proteins. S6. Differential expression of annotated rhoptry (ROP) genes. S7. Differential expression of annotated dense granule (GRA) genes. S8. Differential regulation of TgAP2 genes. [file 12864_2015_1225_MOESM2_ESM.pdf]
